# AP-1 (bZIP) Transcription Factors as Potential Regulators of Metallothionein Gene Expression in *Tetrahymena thermophila*

**DOI:** 10.3389/fgene.2018.00459

**Published:** 2018-10-23

**Authors:** Patricia de Francisco, Francisco Amaro, Ana Martín-González, Juan Carlos Gutiérrez

**Affiliations:** Departamento de Genética, Fisiología y Microbiología, Facultad de Ciencias Biológicas, Universidad Complutense de Madrid, Madrid, Spain

**Keywords:** AP-1 transcription factors, metallothioneins, metal stress, gene expression regulation, *Tetrahymena thermophila*

## Abstract

Metallothioneins (MT) are multi-stress proteins mainly involved in metal detoxification. MT gene expression is normally induced by a broad variety of stimulus and its gene expression regulation mainly occurs at a transcriptional level. Conserved motifs in the *Tetrahymena thermophila* MT promoters have been described. These motifs show a consensus sequence very similar to AP-1 sites, and bZIP type transcription factors might participate in the MT gene expression regulation. In this research work, we characterize four AP-1 transcription factors in each of four different analyzed *Tetrahymena* species, detecting a high conservation among them. Each AP-1 molecule has its counterpart in the other three *Tetrahymena* species. A comparative qRT-PCR analysis of these AP-1 genes have been carried out in different *T. thermophila* strains (including metal-adapted, knockout and/or knockdown strains among others), and under different metal-stress conditions (1 or 24 h Cd^2+^, Cu^2+^, or Pb^2+^ treatments). The possible interaction of these transcription factors with the conserved AP-1 motifs present in MT promoters has been corroborated by protein-DNA interaction experiments. Certain connection between the expression patterns of the bZIP and MT genes seems to exist. For the first time, and based on our findings, a possible gene expression regulation model including both AP-1 transcription factors and MT genes from the ciliate *T. thermophila* has been elaborated.

## Introduction

Metallothioneins (MTs) are multi-stress proteins able to chelate metallic cations through their numerous cysteine residues (Gutierrez et al., 2011; de Francisco et al., 2016). They are considered as multifunctional proteins that participate in different biological processes, such as: maintaining of cellular metal homeostasis, protection against toxic metals (detoxification) or indirect protection against reactive oxygen species (ROS) (Vasak, 2005; Diaz et al., 2007). Although most of the MT genes have certain basal expression levels, their expression is normally induced by a broad variety of stimulus or environmental stressors, such as: metal(loid)s, oxidant and cytotoxic agents, hormones, inflammatory agents and cytokines, growth factors, antibiotics, radiations and other factors considered as stress signals (Haq et al., 2003). Moreover, MT genes expression levels are modified during cell differentiation processes, organism developmental phases (De et al., 1991), cellular aging and some pathologic processes, such as: cancer, Crohn illness, Alzheimer, amyotrophic lateral sclerosis or Wilson and Menkes illnesses (disorders in the copper homeostasis maintaining) (Miles et al., 2000; Laukens et al., 2009; Takahashi, 2015).

Although there are signs of post-transcriptional regulation (Haq et al., 2003), MT gene expression regulation mainly occurs at a transcriptional level by some response *cis* elements located in their promoter regions, where different transcription factors (TFs) can be bound (Gutierrez et al., 2009, 2011). Besides TATA boxes and transcriptional start regions, MT gene promoters can have different motifs related to their expression induction, such as; metal responsive elements (MREs), antioxidant responsive elements (AREs), and glucocorticoid responsive elements (GREs) (Gunther et al., 2012). Moreover, other binding-sites have been detected in some MT gene promoters, as GATA factors binding-sites ([A/T]GATA[A/G]) described in MTs from the nematode *Caenorhabditis elegans* (Moilanen et al., 1999), AP-1 (*Activating Protein-1*) sites (TGA[G/C]TCA) and the GC boxes (GGGGCGGGG) related to the binding of bZIP (*basic region leucine zipper*) TFs (Shaulian and Karin, 2002; Haq et al., 2003; Jenny et al., 2006; Gutierrez et al., 2011; Kumar et al., 2015). A MTCM1 motif (*Metallothionein Conserved Motif 1*) has been described in the promoters of several MT genes from the ciliate *Tetrahymena thermophila*: *MTT1* gene promoter has 6 MTCM1 motifs, *MTT3* only has 2 motifs and *MTT5* has 13 MTCM1 motifs in its duplicated promoter region (Diaz et al., 2007; Gutierrez et al., 2011; de Francisco et al., 2016). In fact, these motifs show a consensus sequence (GTG**TGA**A**TCA**TGAGT) very similar to the previously reported for AP-1 sites and, therefore, it is believed that putative bZIP factors could participate in their gene expression regulation (Diaz et al., 2007; Gutierrez et al., 2011; de Francisco et al., 2016).

The bZIP superfamily is one of the most ancient and conserved eukaryotic TFs analyzed until now (Jindrich and Degnan, 2016). Some of them as Jun/Fos or CREB have been extensively studied in plants and animals and they are used as models to understand DNA-transcription factor interactions (Karin et al., 1997; Jakoby et al., 2002). bZIP TFs are really important due to their participation in multiple biological processes. Moreover, they are involved in the cellular response to different stress situations, such as; the presence of a pathogen, high temperature, osmolarity changes or high salinity levels, the presence of toxic substances, apoptosis, etc. (Karin et al., 1997; Sornaraj et al., 2016; De la Cruz et al., 2017). Although bZIP superfamily includes different types of TFs, all of them are characterized by presenting a highly conserved bZIP motif (60–80 amino acids). This motif is formed by two different adjacent regions: a N-terminal basic region which is usually formed by ∼33 amino acids and allows the binding of TF to the DNA chain and an amphipathic helix or leucine zipper (Karin et al., 1997). The basic region contains a nuclear location signal followed by the conserved motif N-X_7_-R/K (where X is any other amino acid). The leucine zipper is formed by five or more highly conserved leucine residues and, in general, exactly located every 6 amino acids. The bZIP molecules dimerize each other, by the leucine zipper structures, to be able for binding to DNA molecules. In this way, they can form homo- or heterodimers of mutually compatible bZIP proteins according to electrostatic attraction/repulsion processes among polar residues of the leucine helix hydrophobic regions (Karin et al., 1997).

The bZIP superfamily can be grouped into at least seven families (Yang and Cvekl, 2007), and among them AP-1 family includes homo- and heterodimeric forms of Jun (v-Jun, c-Jun, JunB, and JunD) and Fos/Fra (v-Fos, c-Fos, FosB, Fra1, and Fra2) subfamilies. All AP-1 factors are characterized by binding DNA molecules through two possible responsive elements: TRE [*TPA(12-O-tetradecanoate-13-acetate)-responsive element*] which has the consensus sequence **TGA**C**TCA** and CRE (*cAMP-responsive* *el**ement*) whose consensus sequence is **TGA**CG**TCA** (Karin et al., 1997). These transcription factors are induced by different stress conditions (metals, cytokines, H_2_O_2_, etc.) through a cellular signaling pathway where different MAP kinases (JNK, ASK1 and p38, among others) are involved (Karin et al., 1997; Valko et al., 2005). AP-1 expression regulation and activation processes are complex and depend on the dimers formation, possible post-translational modifications and their interactions to specific proteins (protein-kinases, transcriptional coactivators, etc.) (Kappelmann et al., 2014). All these elements define the stability and binding specificity of each AP-1 factor to a particular promoter (Schutze et al., 2008).

AP-1 factors participate in the control of multiple cell processes such as cellular differentiation, proliferation, cancer and apoptosis (Karin et al., 1997; Shaulian and Karin, 2002; Yang and Cvekl, 2007; Shaulian, 2010). They are also involved in gene expression regulation processes in response to different stimulus: cytokines, growth factors, viral or bacterial infections and stress conditions (Durchdewald et al., 2009). Likewise, AP-1 has been related to MT gene expression increasing levels under different stress conditions in different organisms, such as; *Saccharomyces cerevisiae* (Wu et al., 1993; Kumar et al., 2015), *Crassostrea virginica* (Jenny et al., 2006), *Drosophila melanogaster* (Kockel et al., 2001), and *C. elegans* (Moilanen et al., 1999). An increase of *MT-1* metallothionein gene expression level has been related to a higher gene expression of a gene encoding an AP-1 TF in mammalian cells after treatment with an oxidative stressor (Braithwaite et al., 2010). Also, higher *MT-1, c-fos*, and *c-jun* gene expression levels were detected after 24 h copper treatments in simian cells (Mattie et al., 2008).

In this research work, we have carried out a structural *in silico* characterization of four putative AP-1 TFs in the ciliate-model *T. thermophila* (named as TthebZIP1, TthebZIP2, TthebZIP3, and TthebZIP4), and a comparison with AP-1 from other *Tetrahymena* species. Moreover, we have realized an AP-1 gene expression analysis using different *T. thermophila* strains (including metal-adapted, knockout and knockdown strains), under various metal (Cd^2+^, Cu^2+^, or Pb^2+^) treatments (de Francisco et al., 2017, 2018). In addition, experiments to study protein-DNA interactions were carried out, which might confirm the interaction of these AP-1 TFs with MTCM1 motifs present in *T. thermophila* MT gene promoters. Finally, taken together all these data, we have elaborated a model relating all *T. thermophila* metallothionein paralog (*MTT1, MTT3, MTT5*, and *MTT2/4*) gene expression and its presumable regulation by these AP-1 TFs in response to metal-stress.

## Materials and Methods

### Strains and Culture Conditions

Different *T. thermophila* strains were used in this study: SB1969 control strain (*chx1-1/chx1-1*, *mpr1-1/mpr1-1*; pm-S, cy-S, mt II), kindly supplied by Dr. E. Orias (University of California, Santa Barbara, United States), Strains GFPMTT1 and GFPMTT5 harbor multi-copy plasmids bearing the constructs *P_*MTT1*_::GFP::MTT1* or *P_MTT*1*_::GFP::MTT5*, which over-express *MTT1* or *MTT5*, respectively, under the same promoter from *MTT1* gene (Amaro et al., 2014), three metal-adapted strains (Cd-adap, Cu-adap, and Pb-adap) which were obtained after an extensive metal adaptation process and three knockout (KO) and/or knockdown (KD) strains for *MTT1* and/or *MTT5* genes (MTT1KO, MTT5KD, MTT1KO+MTT5KD) (de Francisco et al., 2017, 2018). All these strains were axenically grown in PP210 medium [2% w/v aqueous solution of proteose peptone (Pronadisa), supplemented with 10 μM FeCl_3_ and 250 μg/ml of streptomycin sulfate (Calbiochem) and penicillin G (Sigma)] and maintained at a constant temperature of 30 ± 1°C. Metal-adapted strains were grown with a constant metal pressure equal to their maximum tolerated concentrations (MTC) previously calculated: 115 μM Cd^2+^ (CdCl_2_, Sigma) for Cd-adap strain, 4 mM Cu^2+^ (CuSO_4_⋅5H_2_O, Sigma) for Cu-adap and 5.5 mM Pb^2+^ (Pb(NO_3_)_2_, Sigma) for Pb-adap (de Francisco et al., 2017). To prevent the loss of multi-copy plasmids, GFPMTT1 and GFPMTT5 strains were maintained in 12 μg/ml of paromomycin sulfate (Sigma). Similarly, MTT5KD and MTT1KO+MTT5KD strains were maintained in 800 μg/ml of paromomycin sulfate or 60 μg/ml of cycloheximide, respectively (de Francisco et al., 2017).

### Metal Stress Treatments

The treatment conditions (concentration and time of exposure) for each heavy metal correspond to half the LC_50_ value that results in negligible cell mortality, as were previously determined (Diaz et al., 2007). Cells were exposed for 1 or 24 h to Cd^2+^ (CdCl_2_), Cu^2+^ (CuSO_4_ ⋅ 5H_2_O) or Pb^2+^ [Pb(NO_3_)_2_] (Sigma). SB1969, KO, and KD strains were treated with 44.5 μM Cd^2+^, 315 μM Cu^2+^, or 965 μM Pb^2+^, while metal-adapted, GFPMTT1 and GFPMTT5 strains were exposed to the previously mentioned MTC for each metal. Metal-adapted cells were maintained for 24 h in PP210 medium without any added metal and then were exposed for 1 or 24 h to the MTC of each metal. The MTC-cultures were obtained by continuously culturing cells at their corresponding MTC.

### Total RNA Isolation and cDNA Synthesis

Exponential cell cultures (1–3 × 10^5^ cells/ml) were harvested by centrifugation at 2,800 rpm for 3 min. Total RNA samples were isolated by using the TRIzol Reagent method (Invitrogen). RNA samples were treated with DNase I (Roche) for 30 min at 37°C. RNA integrity was tested by agarose gel electrophoresis and sample concentrations were calculated by the NanoDrop 1000 (Thermo Scientific). MultiScribe Reverse Transcriptase 50 units/μl (Life Technologies) and oligo(dT)-adaptor primer (Roche) were used to synthesize cDNAs from 3.5 μg of total RNA.

### Quantitative RT-PCR (qRT-PCR)

cDNA samples were amplified in duplicate in 96 microtiter plates. Each qPCR reaction (20 μl) contained: 10 μl of SYBR Green (Takara), 0.4 μl of ROX as passive reference dye (Takara), 1 μl of each primer (at 40 nM final concentration), 3.6 μl of ultrapure sterile water (Roche) and 4 μl of a 10^-1^ dilution of cDNA. PCR primers (Supplementary Table S1) were designed using the “Primer Quest and Probe Design” online-application from IDT (Integrated DNA Technologies). β-actin was used as an endogenous control or normalizer gene. Melting curves were obtained and primers specificity was tested by confirming each PCR product by gel electrophoresis and sequencing. Real-time PCR reactions were carried out in an iQ5 real-time PCR apparatus (Bio-Rad) and the thermal cycling protocol was as follows: 5 min at 95°C, 40 cycles (30 s at 95°C, 30 s at 55°C and 20 s at 72°C), 1 min at 95°C and 1 min at 55°C. All controls [no template controls (NTC) and RT minus controls] were negative. Amplification efficiency (E) was measured by using 10-fold serial dilutions of a positive control PCR template. Efficiency parameters were met for all tested genes in all the strains (Supplementary Table S2). Results were finally processed by the standard-curve method (Larionov et al., 2005) and were corroborated with at least two independent experiments, each performed in duplicate. We compared the basal expression levels of different genes using the formula: 2^(Ct1-Ct2)^, being C_t1_ and C_t2_ the cycle threshold (C_t_) values of both genes under a control situation (no metal exposure).

### Protein-DNA Interaction Experiments

Cell extracts were obtained according to the following protocol (Barchetta et al., 2008). From *T. thermophila* cultures ∼10^7^ cells were collected by centrifugation at 1,100 × g during 5 min. The cellular pellet was lysed at 4°C in lysis buffer [10 mM Tris-HCl pH 7.5, 10 mM MgCl_2_ (Sigma), 250 mM sucrose (Panreac), 0.5% Triton X-100 (Merk)], containing 100 μl/ml of a protease inhibitor cocktel (Sigma), 1mM PMSF (Fluka) and 0.5 mM DTT (Sigma). The cellular lysate was centrifugated at 15,000 × g during 10 min at 4°C, and the supernatant discarded. The presence of intact nuclei was detected by staining with propidium iodide and fluorescence microscopy. The obtained pellet was resuspended in 300 μl of cold nuclear protein extraction buffer [50 mM Tris-HCl pH 8, 0.5 M NaCl (PanReac), 10% glycerol (PanReac), 1 mM DTT (Merck), 1 m MPMSF (Sigma), 0.1 mM EDTA (BM)] and incubated at 4°C during 20 min. Then, the cellular debris were eliminated by centrifugation at 15,000 × g during 5 min at 4°C. The supernatant was distributed in 30 μl aliquots immediately frozen under liquid nitrogen and stored at -80°C.

EMSA (*Electrophoretic mobility shift assay*) experiments were carried out according to protocols from Hellman and Fried (2007) and Molloy (2000) with some modifications. All solutions for these assays were performed with Milli Q water (Millipore). In Supplementary Table S3 the oligonucleotide sequences used in EMSA, previously labeled with digoxigenin (DIG High Prime DNA labeling kit) (Roche), are showed. Each one of these oligonucleotides (10 pmoles) were diluted in TEN buffer [0.1 M Tris-HCl pH 8, 1 mM EDTA (BM) pH 8, 50 mM NaCl (PanReac)]. In order to determine the optimal conditions for the protein-DNA binding, different reactions were tested, varying the volumes of each component of the reaction (Supplementary Table S4). The nuclear extract (3–75 μg/μl) was incubated with the digoxigenin-labeled DNA (50 fmoles/μl) in binding buffer [10 mM Tris-HCl pH 7.5, 50 mM NaCl (PanReac), 25 mM MgCl_2_ (Sigma), 4% glycerol (PanReac), 0.5 mM DTT (Merk)] and 1 μg/μl poly(dI-dC). Each reaction (20 μl) was incubated at room temperature (20 min). The binding reaction was stopped adding the 5× electrophoresis sample buffer [300 mM Tris-HCl pH 7.5, 50% glycerol (PanReac), 0.05% bromophenol blue (Bio-Rad), 0.05% xylene cyanol (Bio-Rad)]. The samples were analyzed by polyacrylamide gel electrophoresis [10× TBE buffer (Pronadisa), 40% acrylamide-bisacrylamide, 0.05 g ammonium persulfate (Sigma), 12 ml TEMED (Sigma)], at 10 V/cm (2 h) in 1× TBE buffer. Then, DNA from the gel was electro-blotted onto a nylon^+^ membrane (Roche), at 100 V in 1× TBE buffer, using a Mini Trans-blot Cell system (Bio-Rad), it was bound to the membrane by UV light using a XL-1000 UV crosslinker (Spectronic Corporation). Chemiluminescentnt detection of DNA was performed as described in Detection Started kit II manual (Roche).

To identify nuclear proteins that can interact with the selected DNA fragment we apply the Southwestern blotting method (Labbe et al., 2001; Siu et al., 2008). The nuclear protein extracts were obtained as previously reported in this section. These protein extracts were analyzed by SDS-PAGE (*SDS-polyacrylamide gel electrophoresis*) at 10 V/cm during 2 h (4°C). Then, proteins were electro-blotted onto PVDF membrane (at 150 mA, 2h, 4°C) in electro-blotting buffer [0.25 M Tris, 1.92 M glycine (Sigma), 20% methanol (Merck)], using the Mini-Transblot Cell system (Bio-Rad). Blotted proteins were renaturalized in TNED buffer (10 mM Tris buffer pH 7.5, 50 mM NaCl, 0.1 mM EDTA, 1 mM DTT ] containing 5% skimmed milk powder, and incubated during 4 h at room temperature. After this, the labeled oligonucleotide (50 fmol/μl) in TNED buffer was added and incubated overnight. The membranes were washed in TNED buffer and the labeled probes were chemiluminescent detected as previously reported.

### Partial Protein Purification

The partial protein purification binding to MTCM1 motifs was carried out using the DNA-protein binding purification kit (Roche). The used oligonucleotide (MTCMAB2) presents two repetitions of the MTCM1 motif (Supplementary Table S3). After applying the protocol submitted by the manufacturer, the eluted protein fractions were analyzed by SDS-PAGE and proteins were fixed and stained with a silver staining method (Chevallet et al., 2006).

### Statistical Analysis

Gene expression differences were tested for statistical significance by Student’s *t*-test using the program Statgraphics Centurion XVI (16.1.15 version). *P*-value was fixed in ≤0.05.

### *In silico* Analysis of AP-1 Transcription Factors and Phylogenetic Tree Construction

New putative AP-1 TF sequences were searched in the sequenced genomes of *T. borealis, T. elliotti*, and *T. malaccensis* using the BLAST tool of *Tetrahymena Genome* Database (Stover et al., 2006). *T. thermophila* AP-1 TFs (TthebZIP1, ThebZIP2, TthebZIP3, and TthebZIP4) were used as reference sequences. Access numbers of all the sequences used in this study are collected in Supplementary Table S5. Amino acid composition and molecular weight calculation of new putative AP-1 factors were performed using the online application PIR (*Protein Information Resource*^1^) (Wu et al., 2003). Possible leucine zippers and coiled-coil motifs were identified using two online applications: 2ZIP-Server^2^ (Bornberg-Bauer et al., 1998) and COILS^3^ (Lupas et al., 1991). PSIPRED program^4^ was used to predict secondary structures of bZIP motifs in the AP-1 TFs (Jones, 1999). Possible interactions between coiled-coil regions were calculated by the program bZIP coiled-coil scoring form^5^ (Fong et al., 2004). A multiple sequence alignment was performed using T-Coffee online application (*Tree-based Consistency Objective Function for Alignment Evaluation*) (Notredame et al., 2000). The phylogenetic trees were built with MEGA 5.05 program, using Neighbor Joining algorithm and the bootstrap method with 2,000 repeats. Prediction of three-dimensional (3D) structures was carried out using Phyre2 program (*Protein Homology/Analogy Recognition Engine*^6^) (Kelley et al., 2015) and PyMOL Viewer 1.7.4.5. (PyMOL Molecular Graphics System^7^) was used to visualize them. Putative conserved motifs in 5′UTR regions were described using MEME v.4.11.2 program (*Multiple Em for Motif Elicitation*) (Bailey and Elkan, 1994).

## Results

### Identification and Structural Characterization of AP-1 Transcription Factors in Different *Tetrahymena* Species

**FIGURE 1:**
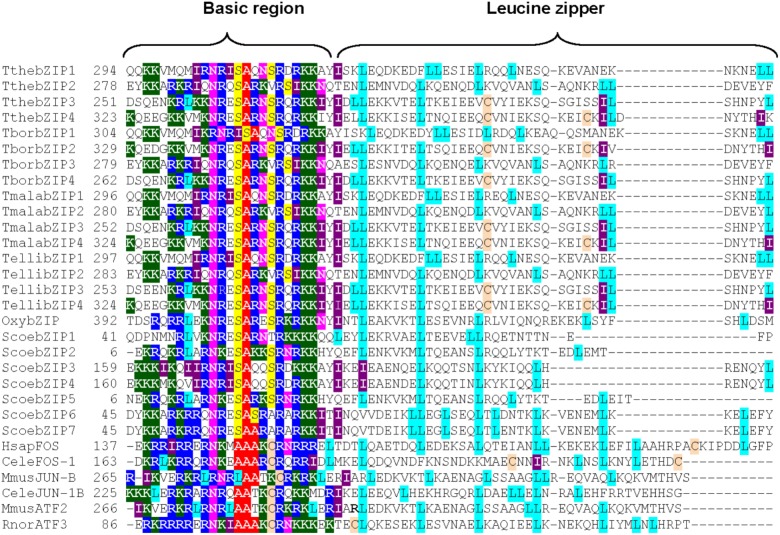
Multiple partial alignment of bZIP motif amino acid sequences from four *Tetrahymena* species and some other model organisms (including two different ciliates). Basic regions and leucine zippers are shown. Conserved residues are marked with different colors: Lys (K) in dark green, Arg (R) in dark blue, Ser (S) in yellow, Asn (N) in fuchsia, Ala (A) in red, Leu (L) in light blue, Ile (I) in violet and Cys (C) in light brown. Tthe, *T. thermophila*; Tbor, *T. borealis*; Tmala, *T. malaccensis*; Telli, *T. elliotti*; Oxy, *Oxytricha* sp.; Scoe, *Stentor coeruleus*; HsapFOS, *Homo sapiens* FOS; CeleFOS-1, *Caenorhabditis elegans* FOS-1; MusJUN-1B, *Mus musculus* JUN-1B; RnorATF3, *Rattus norvegicus* ATF3. Numbers (on the left) indicate the position of the first amino acid shown in the partial alignment. Access numbers of all these sequences are shown in Supplementary Table S5.

Four putative AP-1 genes are detected in the *T. thermophila* macronuclear genome and they have been named; *ThebZIP1*, *TthebZIP2*, *TthebZIP3*, and *TthebZIP4*. Likewise, four putative AP-1 genes have been identified in each of the other three analyzed *Tetrahymena* species (*T. elliotti*, *T. borealis*, and *T. malaccensis*). The main characteristics of these AP-1 TFs are summarized in Table 1. The comparative analysis of these AP-1 shows that each *T. thermophila* AP-1 gene has its matching ortholog in the other three species, sharing common characteristics among them. These genes have introns in a variable number, from only 2 (in *TthebZIP2, TborbZIP3, TmalabZIP2*, and *TellibZIP2*) to 11 (in *TthebZIP3, TborbZIP4, TmalabZIP3*, and *TellibZIP3*) (Table 1). The inferred protein sizes are in the range of 501–746 aa, and we can distinguish two smaller AP-1 proteins (501–595 aa) and two bigger AP-1 proteins (726–746 aa) in each analyzed specie (Table 1). A bZIP motif has been identified in all *Tetrahymena* AP-1 inferred amino acid sequences and they are similar (mainly the basic region) to the bZIP motifs previously described in other AP-1 TFs from different model organisms, such as: *Homo sapiens* (HsapFOS)*, C. elegans* (CeleFOS-1, CeleJUN-1B)*, Mus musculus* (MmusJUN-B) or *Rattus norvegicus* (RnorATF3) (Figure 1). Likewise, we have detected proteins with bZIP motifs in other ciliates; one sequence in *Oxytricha* sp. and seven sequences in *Stentor coeruleus* (Figure 1).

**Table 1:** Structural characteristics of the bZIP genes and inferred proteins from different *Tetrahymena* species.

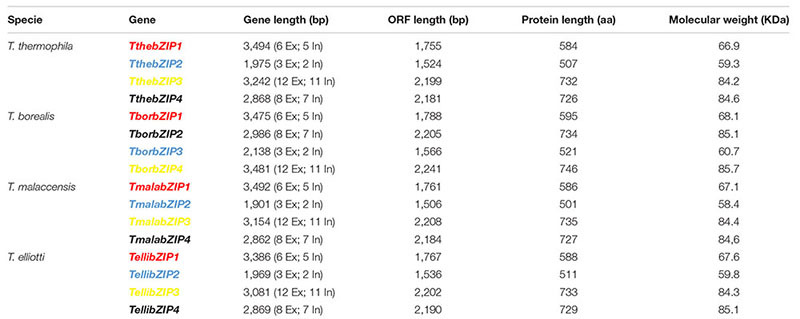

*Colors are used to indicate the corresponding ortholog gene among the four Tetrahymena species. bp: base pairs. Ex, exons; In, introns; aa, amino acids*.

With regard to the leucine zipper region, the majority of *Tetrahymena* AP-1 TFs have the first three leucine (Leu) repeats strictly distributed in its bZIP motifs, and located every 6 or 7 amino acids (Figure 1). This conserved pattern also appears in almost all model organism sequences: HsapFOS, MmusJUN-B or RnorATF3, and in AP-1 from other ciliates (Figure 1). However, the fourth conserved Leu residue, in the majority of the *Tetrahymena* AP-1 TFs, do not appear in the expected position, because the region between the 3rd and 4th Leu in the leucine zipper is considerably greater (13 aa). In only one ortholog of the four AP-1 TFs from each *Tetrahymena* species (indicated in blue in Table 1) there is a Leu residue whose location corresponds to the fourth Leu of the leucine zipper pattern from other organisms (Figure 1). In four other orthologs (bZIP1 in red in Table 1), the 4th Leu residue location matches with the Jun-1B TF from *C. elegans* (Figure 1). In some *Tetrahymena* leucine zipper sequences, the Leu residues are substituted by other aliphatic amino acids like valine (Val) or isoleucine (Ile), constituting a more flexible conformation. This fact is also detected in other bZIP motifs, like FOS1 of *C. elegans* or ATF3 of *R. norvegicus* (Figure 1). Therefore, the *Tetrahymena* leucine zipper pattern contains 5–7 Leu residues or 3–5 Leu plus 1–3 Ile residues, being thus richer in isoleucine residues than other leucine zippers (Figure 1).

Independently of having a strict or flexible leucine pattern, COILS program predicts the presence of one or two potential dimerization motifs in all the *Tetrahymena* AP-1 TF sequences. These motifs are related to the possible formation of coiled-coil structures (Table 2 and Supplementary Figure S1). However, 2ZIP Server program distinguishes between strict or real leucine zippers (only sequences with a strict Leu pattern) and other motifs able to form coiled-coil structures, that allow the dimerization of two proteins but without a strict Leu pattern (Table 2). 2ZIP Server has identified real leucine zippers in the orthologs TthebZIP2, TborbZIP3, TmalabZIP2, TellibZIP2 from different *Tetrahymena* species, and in HsapFOS, MmusJUN-B or RnorATF3 from other selected bZIP TFs (Table 2).

**Table 2:** Identification of real leucine zippers and other motifs related to dimerization processes and coiled-coil structures formation by 2ZIP Server and COILS programs.

Specie	bZIP TF	Presence of strict leucine zipper ^(1)^	Amino acid location	Possible dimerization motif ^(2)^	Amino acid location
*T. thermophila*	TthebZIP1	NO	–	YES (2)	144–178 319–374^∗^
	TthebZIP2	YES (1)	306–334	YES (1)	288–344
	TthebZIP3	NO	–	YES (1)	257–291
	TthebZIP4	NO	–	YES (1)	334–375
*T. borealis*	TborbZIP1	NO	–	YES (2)	146–173 329–384^∗^
	TborbZIP2	NO	–	YES (2)	208–235 339–380^∗^
	TborbZIP3	YES (1)	307–335	YES (1)	290–352
	TborbZIP4	NO	–	YES (1)	268–302
*T. malaccensis*	TmalabZIP1	NO	–	YES (2)	145–179 321–376^∗^
	TmalabZIP2	YES (1)	308–343	YES (1)	291–346
	TmalabZIP3	NO	–	YES (1)	259–293
	TmalabZIP4	NO	–	YES (2)	201–231 335–376^∗^
*T. elliotti*	TellibZIP1	NO	–	YES (1)	322–377
	TellibZIP2	YES (1)	311–339	YES (1)	294–349
	TellibZIP3	NO	–	YES (1)	243–291
	TellibZIP4	NO	–	YES (2)	201–231 335–376^∗^
*H. sapiens*	HsapFOS	YES (1)	165–193	YES (1)	151–193
*C. elegans*	CeleFOS-1	NO	–	YES (1)	188–236
	CeleJUN-1B	NO	–	YES (1)	243–277
*M. musculus*	MmusJUN-B	YES (1)	293–321	YES (1)	263–324
*R. norvegicus*	RnorATF3	YES (1)	114–142	YES (1)	84–145

*^(1)^2ZIP Server program identifies as real leucine zipper only those motifs with a strict leucine pattern. The number of possible leucine zippers is indicated in parentheses for each sequence and its location is also indicated. ^(2)^However, in these sequences with a non-strict leucine pattern, 2ZIP Server identifies possible dimerization motifs. The number of these motifs is indicated in parentheses for each sequence and its location is also indicated. On the other hand, COILS program identifies all the possible regions that could form coiled-coil structures (see also Supplementary Figure S1). Motifs with a higher probability of being dimerization regions (according to COILS program and Figure 1) are marked with an asterisk.*

According to PSIPRED and Phyre2 programs all *in silico* analyzed *Tetrahymena* bZIP TFs have a α-helix located in their putative bZIP motifs. Almost all these proteins have a globular region, including some α-helices. Likewise, Leu residues in leucine zipper are normally disposed in the same rotational positions (data not shown). All *T. thermophila* bZIP motif α-helix models, obtained from the *in silico* 3D analysis, present a 99% of average confidence to different templates; TthebZIP1 or TthebZIP2 (∼99.5% to mafB TF from *H. sapiens*) and TthebZIP3 or TthebZIP4 (∼99.5% to pap1 TF from *Schizosaccharomyces pombe*).

### Prediction of Possible Interactions Between *Tetrahymena* AP-1: Homo- or Heterodimers Formation

Most probable interactions between coiled-coil regions of different AP-1 TFs were predicted by bZIP coiled-coil scoring form program, to know the probability of each AP-1 TF to form homo- or heterodimers. Scores obtained for all possible bZIP interactions for each *Tetrahymena* specie AP-1 are shown in Supplementary Table S6. Higher scores indicate the most probable interactions. Negative values are considered as no optimal interactions. Homodimers formation prevails in all the *Tetrahymena* species for bZIP1, bZIP2, and bZIP3 TFs (Supplementary Table S6). On the other hand, *Tetrahymena* bZIP4 can form homo- or heterodimers independently of the species analyzed (Supplementary Table S6). Specifically, bZIP4 TFs from *T. thermophila*, *T. elliotti*, or *T. malaccensis* have a higher probability to form heterodimers with their corresponding bZIP3 than homodimers with themselves. However, TborbZIP4 has a higher probability to form homodimers than heterodimers with other TborbZIP TF (Supplementary Table S6). In this way, according to the scores obtained, the following heterodimers could be formed: TthebZIP3/4, TborbZIP2/4, TellibZIP3/4, and TmalabZIP3/4 (Supplementary Table S6). In contrast, some TF don’t seem to have an optimal bZIP motif to interact with other different bZIP motifs, such as: TthebZIP2, TborbZIP3, TellibZIP1, TellibZIP2, and TmalabZIP2, that obtain negative or low scores when they are put together with other bZIP factors (Supplementary Table S6).

### Identification of Conserved Motifs in Putative Promoter Regions of *T. thermophila* AP-1 Genes

To know if the expression of *T. thermophila* AP-1 genes could be jointly regulated, an *in silico* analysis of their putative promoter regions was carried out. Three conserved motifs have been identified in 5′UTR regions from all *T. thermophila* AP-1 genes (Table 3). Probably, these motifs may be involved in the gene expression regulation of these AP-1 genes, with the following consensus sequences: GCT(N)_3_CT for motif 1, G(S)_2_TT(B)_2_C for motif 2 and (H)_2_C(R)A(V)T(N)_4_C(N)_5_A(N)_3_T(N)_3_A(M)_2_A for motif 3 (letters other than C,G,A or T follow the IUPAC nucleotide code).

**Table 3:** Conserved motifs located in putative promoter regions of the four *T. thermophila* AP-1 genes.

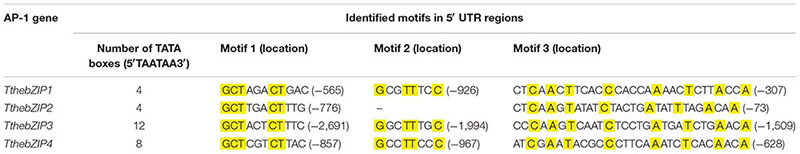

*No detected: (-). Motif sequence locations (in parentheses) for the three conserved motifs are shown. Common residues among the four AP-1 sequences are shaded in yellow*.

One copy of each motif has been located in each AP-1 gene promoter, excepting for the motif 2 that is not present in the *TthebZIP2* gene (Table 3). Motif 2 has the highest percentage of conserved residues (50%) among the four AP-1 sequences, followed by motif 1 (45%) and, finally, by motif 3 (28%). In all AP-1 genes, motif 3 is the nearest located to the beginning of the ORF (Table 3). Likewise, motif 3 is the longest (29 bp) in comparison to the other two motifs with 11 (motif 1) and 8 bp (motif 2).

### Comparative Analysis of *T. thermophila* AP-1 Gene Expression Induction Under Different Metal Stress Treatments

**FIGURE 2:**
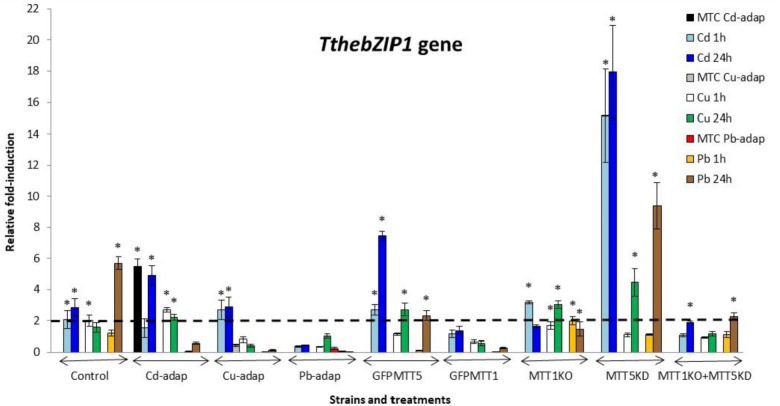
*TthebZIP1* gene induction levels after Cd^2+^, Cu^2+^, or Pb^2+^ treatments (1 or 24 h) from different *Tetrahymena thermophila* strains. MTC, maximum tolerated concentrations by metal-adapted strains: 115 μM Cd^2+^, 4 mM Cu^2+^, and 5.5 mM Pb^2+^. Each histogram bar represents an average value ± standard deviation (see Supplementary Table S7) from two or three independent experiments. Relative induction values are represented in a logarithmic scale. Asterisks indicate significant differences from the control with *p* < 0.05.

**FIGURE 3:**
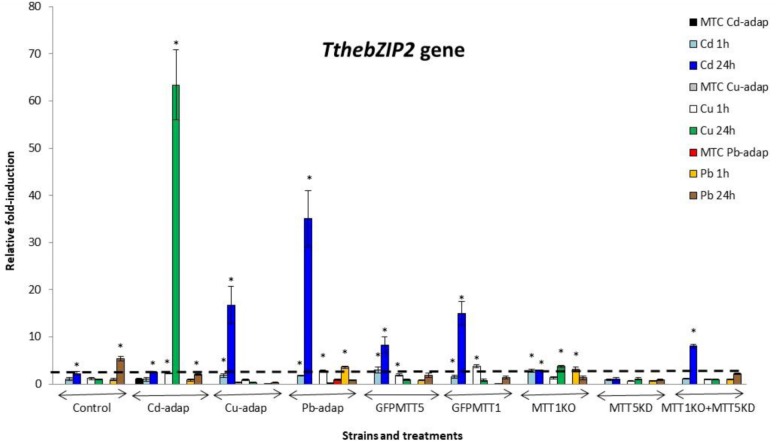
*TthebZIP2* gene induction levels after Cd^2+^, Cu^2+^, or Pb^2+^ treatments (1 or 24 h) among different *T. thermophila* strains. MTC, maximum tolerated concentrations by metal-adapted strains: 115 μM Cd^2+^, 4 mM Cu^2+^, and 5.5 mM Pb^2+^. Each histogram bar represents an average value ± standard deviation (see Supplementary Table S7) from two or three independent experiments. Relative induction values are represented in a logarithmic scale. Asterisks indicate significant differences from the control with *p* < 0.05.

**FIGURE 4:**
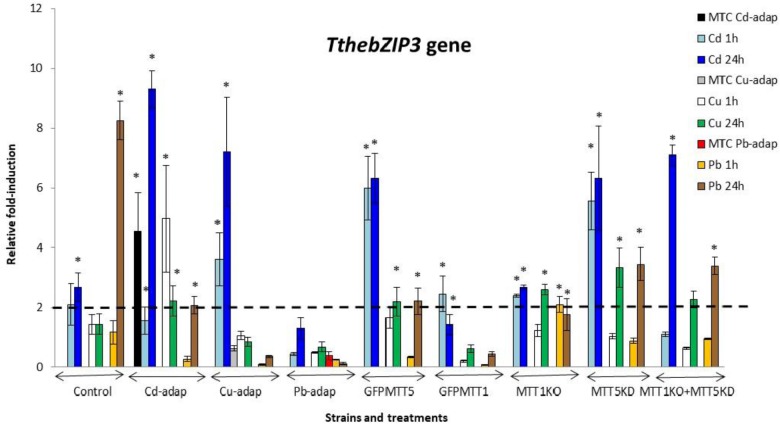
*TthebZIP3* gene induction levels after Cd^2+^, Cu^2+^, or Pb^2+^ treatments (1 or 24 h) among different *T. thermophila* strains. MTC, maximum tolerated concentrations by metal-adapted strains: 115 μM Cd^2+^, 4 mM Cu^2+^, and 5.5 mM Pb^2+^. Each histogram bar represents an average value ± standard deviation (see Supplementary Table S7) from two or three independent experiments. Relative induction values are represented in a logarithmic scale. Asterisks indicate significant differences from the control with *p* < 0.05.

**FIGURE 5:**
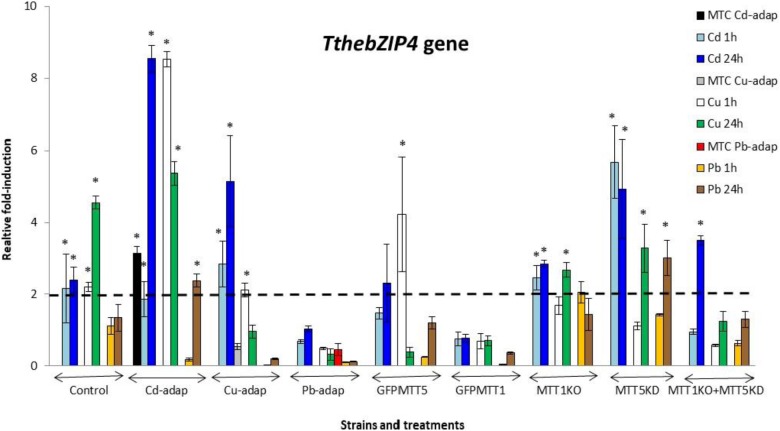
*TthebZIP4* gene induction levels after Cd^2+^, Cu^2+^, or Pb^2+^ treatments (1 or 24 h) among different *T. thermophila* strains. MTC, maximum tolerated concentrations by metal-adapted strains: 115 μM Cd^2+^, 4 mM Cu^2+^, and 5.5 mM Pb^2+^. Each histogram bar represents an average value ± standard deviation (see Supplementary Table S7) from two or three independent experiments. Relative induction values are represented in a logarithmic scale. Asterisks indicate significant differences from the control with *p* < 0.05.

The relative gene expression induction levels (calculated by qRT-PCR) for the four AP-1 genes from *T. thermophila*, after 1 or 24 h treatments with three different heavy metals (Cd^2+^, Cu^2+^, or Pb^2+^), have been analyzed and compared. This analysis has been performed with different strains of this *Tetrahymena* species (see Experimental section) and results are shown in Figures 2–5 and Supplementary Table S7.

*TthebZIP1* gene is mainly induced under Cd^2+^ treatments and, in general, higher induction levels are detected after 24 h exposures (Figure 2). Specifically, the highest induction values were observed for MTT5KD, GFPMTT5, and Cd-adap strains. On the other hand, this gene is also induced under 24 h Pb^2+^ treatments (mainly, in control and MTT5KD strains) or, even, under 24 h Cu^2+^ treatments (in GFPMTT5, MTT1KO, and MTT5KD strains) but in this case a lower expression level is detected. Therefore, the induction ranking for *TthebZIP1* gene is: Cd > Pb > Cu (Figure 2 and Supplementary Table S7).

In general, *TthebZIP2* is the *T. thermophila* AP-1 gene that achieves the highest induction level values (Figure 3). Just like *TthebZIP1*, higher induction values are observed after 24 h treatments. It is enormously highlighted the high induction value obtained under 24 h Cu^2+^ treatments in the Cd-adap strain. On the contrary, this gene is mainly induced under 24 h Cd^2+^ treatments in Cu-adap, Pb-adap, GFPMTT5, GFPMTT1, and MTT1KO+MTT5KD strains or even after 24 h Pb^2+^ exposures in the SB1969 control strain, although this value is considerably lower (Figure 3). *TthebZIP3* (Figure 4) and *TthebZIP4* (Figure 5) genes present, in general, lower induction levels (<10×) than the other two AP-1 genes previously analyzed. *TthebZIP3* is mainly induced after Cd^2+^ (1 or 24 h) treatments in Cd-adap, Cu-adap, GFPMTT5, MTT5KD, and MTT1KO+MTT5KD strains and after 24 h Pb^2+^ exposures in control, MTT5KD, MTT1KO+MTT5KD strains (Figure 4 and Supplementary Table S7). Likewise, certain induction levels are detected after Cu^2+^ treatments in the case of Cd-adap (1 h) and MTT5KD (24 h). *TthebZIP4* has a similar induction pattern to *TthebZIP3* under Cd^2+^ exposures, but it is also considerably induced under Cu^2+^ treatments (1 or 24 h) in Cd-adap, GFPMTT5, MTT5KD and control strains (Figure 5 and Supplementary Table S7).

In Supplementary Figure S2, a comparative analysis of the different AP-1 gene expression patterns obtained from each analyzed *T. thermophila* strain and under different metal exposures (including basal expression levels and induction values > 2) is showed. *TthebZIP2* gene expression normally appears in the first positions in almost all analyzed strains, independently of the metal treatment. However, a clear exception is detected in MTT5KD strain, where none metal treatment obtains significant induction values for *TthebZIP2 gene* (Supplementary Figure S2). On the contrary, *TthebZIP1* gene occupies the first position of the ranking in this knockdown strain for all metal treatments: bZIP1 ≥ bZIP3 ≥ bZIP4 (Supplementary Figure S2). On the other hand, *TthebZIP4* gene expression is normally located in the last positions of the ranking after Cd^2+^ or Pb^2+^ exposures, but it holds the first or second position under Cu^2+^ treatments (Supplementary Figure S2). In Cu or Pb-adap strains, only one AP-1 gene is significantly induced in response to a Cu^2+^ or Pb^2+^ exposure, and *TthebZIP2* and *TthebZIP4* genes are involved in this “unique response” situation. Finally, *TthebZIP1* and *TthebZIP3* genes present a less-defined location into the different induction patterns, presenting a higher variability. However, *TthebZIP3* achieves the highest induction values under lead treatments, reaching the first positions of the ranking in some *Tetrahymena* strains, while it occupies more remote positions under copper exposures (Supplementary Figure S2).

By way of summary, we can also analyze separately each *Tetrahymena* strain and compare its AP-1 gene induction values among them:

1. SB1969 control strain shows similar induction patterns for *TthebZIP1*, *TthebZIP2*, and *TthebZIP3* genes, which mainly respond to Pb^2+^ and Cd^2+^ (24 h treatments) (Figures 2–5). *TthebZIP4* has a very different pattern and it is mainly induced by Cu^2+^ and Cd^2+^ (24 h treatments) (Figure 5).
2. *TthebZIP2* is the highest induced AP-1 gene in Cd-adap strain in response to Cu^2+^ (24 h) (Figure 3). *TthebZIP3* and *TthebZIP4* have a very similar induction pattern, responding mainly to Cd^2+^ (24 h) and Cu^2+^ (1 h) (Figures 4, 5). *TthebZIP1*in this strain is mainly expressed under Cd^2+^ (24 h) (Figure 2).
3. Likewise, *TthebZIP2* genes stands out in Cu-adap strain and it shows the highest induction values under 24 h Cd^2+^ treatment (Figure 3). The other three *AP-1* genes show lower induction values and similar patterns among them, being also the highest induction values under 24 h Cd^2+^ treatments (Figures 2–5).
4. *TthebZIP2* gene is the only one that shows significant induction values in Pb-adap strain. It achieves the highest induction values under 24 h Cd^2+^ treatment (Figure 3).
5. In GFPMTT5 strain, *TthebZIP1*, *TthebZIP2*, and *TthebZIP3* genes present similar induction patterns, and the highest induction values are obtained under 24 h Cd^2+^ treatments (Figures 2–5). On the other hand, *TthebZIP4* gene has a very different pattern and it is mainly expressed after 1 h Cu^2+^ treatment (Figure 5).
6. GFPMTT1 strain has a similar AP-1 gene expression pattern to Pb-adap strain as *TthebZIP2* is the only one that responds to metal-treatments; this gene is mainly induced after 24 h Cd^2+^ exposure (Figure 3).
7. Finally, regarding KO and/or KD strains, MTT5KD is the strain that shows the highest AP-1 gene induction levels: *TthebZIP1* is mainly induced under Cd^2+^(1 or 24 h), Cu^2+^or Pb^2+^ (24 h treatments) (Figure 2), *TthebZIP3* and *TthebZIP4* genes have similar patterns and they achieve the highest induction values after Cd^2+^ treatments (Figures 4, 5) and *TthebZIP2* doesn’t show significant induction values for any metal treatment. On the other hand, *TthebZIP2* and *TthebZIP3* are the AP-1 genes that better responds in MTT1KO+MTT5KD strain under Cd^2+^ (24 h) exposures (Figures 3, 4). Finally, low induction values have been detected for every AP-1 gene in the MTT1KO strain (Figures 2–5).

### *T. thermophila AP-1* Gene Basal Expression Levels: A Comparative Analysis

C_t_ values obtained by qRT-PCR under control conditions (no metal treatments) (Supplementary Table S8) have been compared among the four AP-1 genes in each *Tetrahymena* strain (Table 4 and Supplementary Figure S2) and among the different strains for each AP-1 gene (Table 5). In all analyzed strains, *TthebZIP4* gene shows the highest basal expression levels whereas *TthebZIP3* exhibits the lowest values (Supplementary Figure S2). Therefore, the consensus AP-1 gene basal expression pattern for almost all analyzed *T. thermophila* strains is: *bZIP4* > *bZIP1* > *bZIP2* > *bZIP3* (Supplementary Figure S2). The highest differences between *TthebZIP4* and *TthebZIP3* basal expression levels are detected in GFPMTT5 (∼ 32×) and SB1969 control (∼ 27×) strains (Table 4). On the other hand, *TthebZIP1* and *TthebZIP2* genes show quite similar basal expression levels in the majority of the *T. thermophila* strains, excepting for MTT5KD strain, in which *TthebZIP2* expression level is ∼5× higher than *TthebZIP1* gene (Table 4). *TthebZIP1* and *TthebZIP2* gene basal expression levels are usually higher than those showed by *TthebZIP3* gene, regardless of the considered strain (Table 4 and Supplementary Figure S2).

**Table 4:** Comparison of basal expression levels among the four AP-1 genes in each analyzed *T. thermophila* strain.

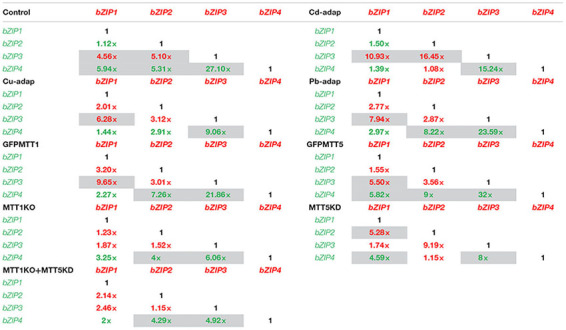

*AP-1 genes basal expression levels are compared in each T. thermophila strain, distinguishing them by two colors: red and green. For each comparison, results are indicated in red or green depending on the AP-1 gene that has a higher basal expression level in the same strain. Comparison values higher than 4 (C_t_ value differences higher than 2 cycles) are shaded in gray.*

**Table 5:** Comparison of basal expression levels for each AP-1 gene among different *T. thermophila* strains.

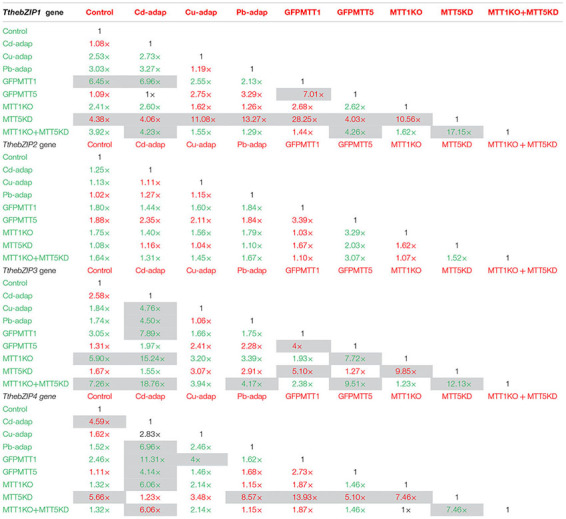

*Different T. thermophila strains are compared by twos, distinguishing them in two colors: red and green. For each comparison, results are indicated in red or green depending on the strain that has a higher basal expression level for the same AP-1 gene. Comparison values higher than 4 (C_t_ value difference higher than 2 cycles) are shaded in gray.*

The comparison of basal expression levels for each AP-1 gene between the control (SB1969) and the other strains (Table 5), reveals that, in general, *TthebZIP2* levels are not significantly increased in none of the analyzed strains. *TthebZIP1* basal expression levels are increased in GFPMTT1 (∼6.5×) and *TthebZIP3* shows higher basal expression levels in MTT1KO (∼6×) and MTT1KO+MTT5KD (∼7×) than control strain (Table 5). Comparing *AP-1* basal expression levels among all the analyzed strains, we detect that GFPMTT1 strain is the one that has the highest basal expression levels for *TthebZIP1*, *TthebZIP2*, and *TthebZIP4* genes. Likewise, *TthebZIP3* is considerably expressed in MTT1KO and MTT1KO+MTT5KD strains (Supplementary Table S8). In contrast, the lowest basal expression levels are detected in the following strains: MTT5KD (*TthebZIP1* and *TthebZIP3*), GFPMTT5 (*TthebZIP3*) and Cd-adap (*TthebZIP3*) (Supplementary Table S8).

Summarizing, *TthebZIP1* gene presents the highest basal expression levels in GFPMTT1 and MTT1KO+MTT5KD strains and its lowest levels in MTT5KD (Supplementary Table S8). *TthebZIP2* shows very similar basal expression levels in all the analyzed strains. The highest *TthebZIP3* basal expression levels are detected in MTT1KO+MTT5KD, MTT1KO and GFPMTT1 strains, while its lowest levels are seen in Cd-adap, GFPMTT5 and MTT5KD strains (Supplementary Table S8). Finally, the highest basal expression levels in *TthebZIP4* gene are detected in GFPMTT1, MTT1KO, MTT1KO+MTT5KD, and Pb-adap strains and its lowest levels were in Cd-adap and MTT5KD strains (Supplementary Table S8).

### Analysis of Nuclear Protein-MTCM1 Motif Interactions

To find out if there are nuclear proteins that can be attached to the MTCM1 motif, several EMSA tests were carried out. The digoxigenin-labeled MTCM1 oligonucleotide was incubated at room temperature during 30 min under increasing concentrations of *T. thermophila* macronuclear protein extracts and different reaction conditions (Supplementary Figure S3). As an unspecific competitor, the poly(dI-dC) oligonucleotide was added to the reaction. Results have shown that two different majority protein bands are present (Supplementary Figure S3A), indicating that, at least, several putative nuclear proteins can bind to this DNA motif. The band intensity and, therefore the amount of DNA-protein complex formed, was higher as the concentration of the nuclear extract increased (Supplementary Figure S3A). In addition, after using nuclear extracts from *T. thermophila* Cd-treated cultures (0.9 mM, in Tris-HCl buffer), during 2 h (Supplementary Figure S3B), bands with a higher intensity, with regard to samples exposed during 30 min, are detected (Supplementary Figure S3B). Which indicates that after a longer Cd-treatment a larger amount of DNA-bound protein is present. The specificity of DNA-binding protein was evidenced by the addition of non-digoxigenin-labeled DNA (specific competitor at 50×, 100×, or 200× concentration), which significantly reduced the labeled DNA-protein complex formation (Supplementary Figure S3B). The addition of Zn^2+^ does not increase the formation of the DNA-protein complex, so it seems that the proteins binding to the MTCM1 motif are not Zn finger proteins. Both negative controls (Supplementary Figure S3) reveal that the MTCM1 motif-binding protein is exclusively from *T. thermophila*.

Using the Southwestern blotting technique we tried to get to know the molecular weight of the proteins that bind to the MTCM1 motif. The Supplementary Figure S4 shows that, at least, three different bands are detected, indicating that the digoxigenin-labeled MTCM1 oligonucleotide can interact with, at least, three different proteins present in the *T. thermophila* nuclear extract. Their estimated molecular weights are in the range; 30–57, 57–93, and 93–116 KDa, respectively (Supplementary Figure S4). The inferred molecular weights of the putative *T. thermophila* AP-1 proteins (Table 1); TthebZIP1 (66.9 KDa), TthebZIP2 (59.3 KDa), TthebZIP3 and TthebZIP4 (∼84 KDa), are in or near of any of these ranges. No bands are detected in the negative control (Supplementary Figure S4, lane C), thus discarding the possibility that the detected bands (lanes 1 and 2 in Supplementary Figure S4) are due to an unspecific binding of the MTCM1 oligonucleotide to any protein.

After verifying that one or more proteins from the *T. thermophila* nuclear extract interact with the MTCM1 motif, we have tried to purify some of these proteins. The eluted fractions, containing presumably the proteins retained by the MTCM1 oligonucleotide, were analyzed by SDS-PAGE. Several bands with molecular weights within the range 40–85 KDa were detected (data not shown). Unfortunately, the amounts obtained of these proteins (only detected after silver staining) were not sufficient for their identification.

## Discussion

### Identification and Characterization of Four AP-1 Genes in *Tetrahymena* Species: Existence of a High and Significant Numerical and Structural Conservation

After examining the sequenced macronuclear genomes of four *Tetrahymena* species (*T. thermophila, T. borealis, T. elliotti*, and *T. malaccensis*), we have found four putative bZIP transcription factors in each species. Each of the four bZIP genes has its corresponding counterpart in the other three *Tetrahymena* species (Table 1). TthebZIP1 is very similar to TmalabZIP1 (95%), TellibZIP1 (88%), and TborbZIP1 (71%) (Supplementary Table S9). TthebZIP2 shares high identity percentages with TmalabZIP2 (82%), TellibZIP2 (75%), and TborbZIP3 (58%). TthebZIP3 is very similar to TmalabZIP3 (94%), TellibZIP3 (89%), and TborbZIP4 (80%), and TthebZIP4 shares high identity percentages with TmalabZIP4 (95%), TellibZIP4 (89%), and TborbZIP2 (79%) (Supplementary Table S9). Something similar happens between yeast species, such as; *S. cerevisiae* and *Kluyveromyces lactis*, which have AP-1 ortholog genes with a high similarity (Billard et al., 1997).

**FIGURE 6:**
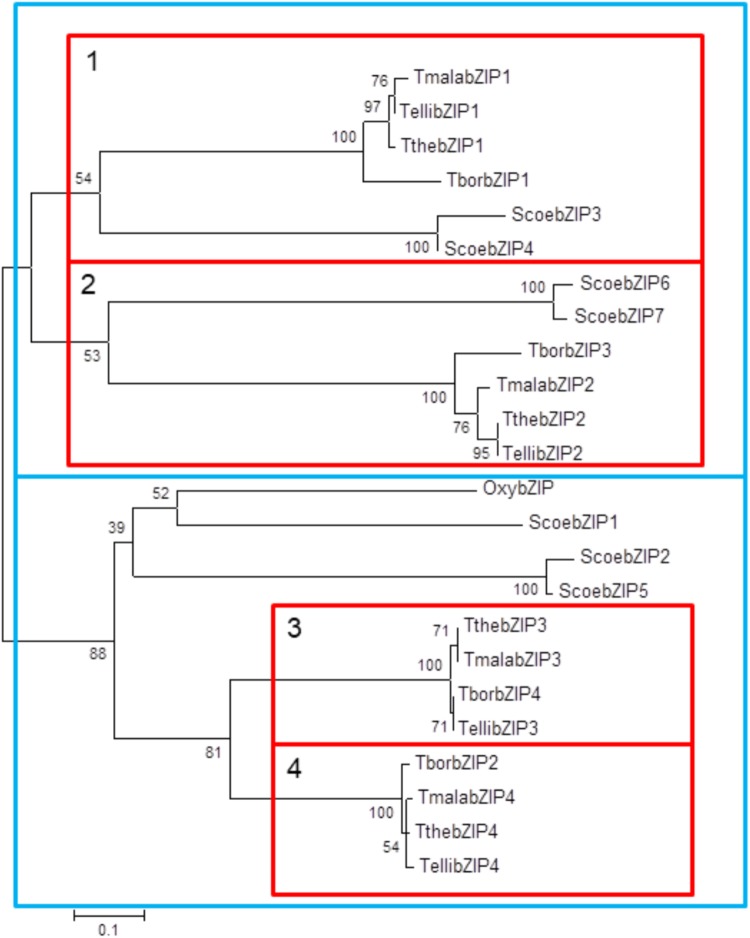
Phylogenetic tree of bZIP amino acid sequences from different ciliates. Each branch length follows the scale. Calculated bootstrap values are indicated in each node. Tthe, *T. thermophila*; Tbor, *T. borealis*; Telli, *T. elliotti*; Tmala, *T. malaccensis*; Oxy, *Oxytricha* sp.; Scoe, *Stentor coeruleus*. Red boxes include the four bZIP subgroups (1–4), each one composed by four bZIP TFs from different *Tetrahymena* species. Blue boxes are grouping these four groups into two major groups depending on the size range of their bZIP proteins; large (726–746 aa) and small (501–595 aa).

These high identity percentages are also reflected in the phylogenetic tree showed in Figure 6. All *Tetrahymena* bZIP protein sequences are divided into two large groups, each of which is subdivided into two groups that include one of the each homologous bZIP protein. Although the four *Tetrahymena* species belong to *Borealis* group (Chantangsi and Lynn, 2008), *T. thermophila* and *T. malaccensis* (both included in RSA1 riboset) are the nearest from a phylogenetic point of view, and, likewise, their AP-1 transcription factors are those with the highest identity percentages. On the other hand, *T. borealis* (RSA2 riboset) (Chantangsi and Lynn, 2008) is the furthest specie from a phylogenetic point of view and its AP-1 transcription factors show, in general, the lowest identity percentages comparing to the others. In addition, conservation among *Tetrahymena* bZIP transcription factors is not only numerical (four AP-1 genes in each *Tetrahymena* species) but also structural, as the four AP-1 TFs that form each group share very similar structural features, such as: the length of their amino acid sequences, the number of exons and introns in their corresponding genes (Table 1) or the location of their dimerization motifs (Table 2). In fact, these four groups are grouped into two major groups in the phylogenetic tree (Figure 6), depending on the size range of their bZIP proteins; one group formed by larger (726–746 aa) and another group formed by smaller bZIP proteins (501–595 aa).

Four of the bZIP sequences from *S. coeruleus* are included in the first large group (smaller bZIP molecules); two are close to the first subgroup of *Tetrahymena* bZIP proteins and two are related to the second subgroup of *Tetrahymena* bZIP proteins. Three bZIP of *S. coeruleus* are related to the second large group (larger bZIP molecules), but separated from those of *Tetrahymena*. Also, the only bZIP protein from *Oxytricha* sp. is related to ScoebZIP1 protein, from *S. coeruleus*, in the second large group of ciliate bZIP TFs (Figure 6).

All *Tetrahymena* AP-1 TFs have the characteristic bZIP motif and all of them share a high level of conservation, and this assertion can be extended to all bZIP proteins found in ciliates (Figure 1). Their basic regions, that allow DNA binding, show many conserved residues among very different organisms including the 24 ciliate analyzed sequences. Specifically, the following motif stands out: NR/KxSAxx(N/K)(S/V)Rx(R/I)KK, where the underlined amino acid residues are the most conserved, and x means other amino acids. The high similarity of the bZIP basic region among different organisms suggests a common origin from an ancestral eukaryotic bZIP, which would include the highly conserved NxxSAxxSR signature motif. This DNA binding motif has not changed in the course of the bZIP superfamily evolution. Using 49 sequenced genomes from different eukaryotic groups, the evolution of the oldest family of eukaryotic transcription factors (bZIP) has been reconstructed, and authors have demonstrated that the evolution of multicellularity was paralleled to the expansion and diversification of bZIP genes in different families (Jindrich and Degnan, 2016). It has been proposed that the bZIP TF from the pathogenic flagellated protozoon *Giardia lamblia* could be the precursor of all bZIPs (Amoutzias et al., 2007). A phylogenetic analysis including bZIP TFs from very diverse organisms (including *Tetrahymena* and other protists) (data not shown), reveals that both TthebZIP3 and TthebZIP4 are close to *Giardia* bZIP, and so we could also consider them phylogenetically near.

Leucine zippers also have a high conservation level, but this one is lower than the previously mentioned for the basic regions. Leucine-rich regions allow dimerization processes (Hurst, 1995) and, probably for this reason, Leu residues are usually exposed to the surface of the protein, allowing the binding of two bZIP monomers and the obtaining of active conformations (homo- or heterodimers) which will regulate the expression of specific genes. *Tetrahymena* leucine zipper patterns contain 5–7 Leu residues like other bZIP motifs, but they are richer in isoleucine residues than other leucine zippers (including those from other ciliates). In *Tetrahymena* larger protein AP-1 group (mainly bZIP3 and bZIP4), unlike what happens in the majority of bZIP motifs and like it occurs in some Fos TFs (from *C. elegans* or *H. sapiens*), 1 or 2 conserved cysteine (Cys) residues are present in the leucine zipper (Figure 1). In *Tetrahymena* bZIP3 and bZIP4 proteins, one of these Cys residues is located in the same place that the third conserved leucine residue (Figure 1). We can speculate about the possibility that SH- groups from Cys residues could facilitate, after binding to a metallic cation (Cd^2+^ or another), the homo- or hetero-dimerization of two AP-1 monomers, forming homodimers (bZIP3/3 or bZIP4/4) or heterodimers (bZIP3/4). Thus, the presence of a toxic metal could induce the dimerization of AP-1 monomers, and induce the expression of metallothionein genes. In the yeast *S. cerevisiae* an AP-1 TF (Yap8) is critical for arsenic tolerance, and the authors have demonstrated that Yap8 directly binds to As^3+^ (one arsenite ion per one Yap8 monomer) (Kumar et al., 2015). The binding site is constituted by three Cys residues present in Yap8, and it does not require an additional protein. Binding of As^3+^ to Yap8 induces a conformational change that converts the inactive Yap8 form into an active transcriptional regulator. Therefore, Yap8 DNA-bound form acts as an As^3+^ molecular sensor.

Strict leucine zippers have been detected in only one AP-1 ortholog of each of the four *Tetrahymena* species (Table 2), which are grouped in the same subgroup present into the smaller bZIP protein group shown in Figure 6. The rest of *Tetrahymena* AP-1 orthologs, even if they do not have a strict leucine zipper, are able to carry out homo- or hetero-dimerization.

The *in silico* inferred 3D structure analysis reveals that in almost all *Tetrahymena* bZIP proteins some others α-helices attached to the α-helix of the bZIP motif exist, which are probably eliminated after a post-translational processing to originate the functional monomer, or this protein region facilitates in some way their dimerization.

The probability of each *Tetrahymena* AP-1 TF to form homo- or heterodimers was tested (Supplementary Table S6). The phylogenetic subgroups including different *Tetrahymena* bZIP1 and bZIP2 (subgroups 1 and 2 in Figure 6) preferentially form homodimers, while heterodimers are preferably formed between bZIP3 and bZIP4 molecules (subgroups 3 and 4 in Figure 6) of each corresponding *Tetrahymena* species: TthebZIP3/4, TellibZIP3/4, and TmalabZIP3/4. However, although heterodimers may be formed in *T. borealis* (TborbZIP2/4), the monomer TborbZIP4 is more likely to form homodimers than heterodimers with other TborbZIP monomers.

The products of these ortholog and highly conserved genes may perform similar functions in the different *Tetrahymena* species, just like has been reported in some yeasts, where complementation studies have been carried out using mutant strains for AP-1 genes. It has been checked that ortholog genes from other species can complement AP-1 mutations in another species (Billard et al., 1997). Furthermore, if we compare the different bZIP isoforms of each *Tetrahymena* species, we observe low identity percentages among them (22–45%) (Supplementary Table S9). This fact may corroborate the hypothesis that the four isoforms are necessary in each species as they have different functions among them. In addition, for each *Tetrahymena* species, the two bZIP isoforms with a higher identity percentage (43–45%) (Supplementary Table S9) are also the ones with a high probability of forming heterodimers.

After an analysis of conserved motifs in the putative promoter regions of *Tetrahymena* AP-1 genes, we have found three conserved DNA motifs (Table 3) and one copy of each motif is present in almost all *Tetrahymena* AP-1 5′UTR regions, which corroborates the hypothesis that these motifs may be involved in the expression regulation of these genes. By presenting the same regulatory motifs, we could think about the existence of a coordinated expression between the different ortholog genes.

### Nuclear Protein-MTCM1 Motif Interactions

*Electrophoretic mobility shift assay* analysis have showed that, at least one protein, or probably several proteins from the *T. thermophila* macronuclear extract interacts with the MTCM1 motif. Because of its similarity with the molecular weight of some of them, it could be identified with some of the TthebZIP TFs. But, unfortunately, the obtained amounts of these proteins were not sufficient for their identification. Therefore, the only thing we can infer from these experiments is that these DNA conserved motifs, present in the *T. thermophila* MT gene promoters, can be linked to proteins acting as transcription factors.

### Putative Functional Attributions to the Different *T. thermophila* AP-1 Transcription Factors

According to qRT-PCR results, the four *T. thermophila* bZIP TFs may have different and complementary functions in response to a stressful situation. In general, transcription factors act in low concentrations, and the expression levels of their encoding genes do not achieve high values. Therefore, small detected changes are significantly considered because a slightly increase in their expression levels is enough to perform their biological functions (Kouno and Ezaki, 2013). However, there are exceptions such as some TFs from the bacterium *Helicobacter pylori* under different environmental conditions (De la Cruz et al., 2017). Likewise, we have detected relatively high induction levels for some *Tetrahymena* AP-1 genes, under specific experimental conditions and in diverse strains. In this way, *T. thermophila* AP-1 TFs seem to have a key role as expression activators for genes related to metal-stress responses like MT genes, and probably to other environmental stressors. It is difficult to relate the expression profiling of these bZIP genes to each MT gene or to assign a specific bZIP TF to a particular MT gene expression. In the following sections we try to extract, from our actual and previous results, the clearest possible connections between the different bZIP TFs and the MT gene expression inductions from *T. thermophila* under different metal stress conditions.

### TthebZIP1: A Probable Transcription Factor for *MTT1* Gene Expression

In all analyzed strains, *TthebZIP1* gene shows, under no metal-treatment, the second place in the ranking of basal expression values of *T. thermophila* AP-1 genes (Supplementary Figure S2). However, although it is in second place in the ranking, in GFPMTT1 strain this gene has a considerably higher basal expression level than control strain (SB1969): ∼6.5× and ∼3× and ∼10× regarding to *TthebZIP2* and *TthebZIP3* basal expression levels, respectively. GFPMTT1 strain has a higher number of copies for the *MTT1* gene (Amaro et al., 2014) and it shows the highest *MTT1* basal expression level (de Francisco et al., 2017). Likewise, in GFPMTT5 (which has the same *MTT1* gene promoter than the strain GFPMTT1) (Amaro et al., 2014), the AP-1 basal expression ranking is the same (Supplementary Figure S2), and, like GFPMTT1, it shows the highest *MTT1* basal expression level (de Francisco et al., 2017). Therefore, higher *TthebZIP1* gene expression levels may be required in these strains for maintaining some MT gene basal expression levels, such as *MTT1* levels, as this gene normally shows the highest basal expression levels under no metal exposures (de Francisco et al., 2017). In this way, artificially increased *MTT1* levels may also cause an increase of *TthebZIP1* basal expression levels in the GFPMTT1 or GFPMTT5 strains.

Likewise, *TthebZIP1* gene induction patterns under metal exposures are similar to the ones previously described for *MTT1* gene (de Francisco et al., 2017): the highest induction values are detected after 24 h Cd^2+^ treatments and, in some strains, also after 24 h Pb^2+^ exposures (Figure 2). In general, higher *TthebZIP1* induction values are observed after 24 h treatments, so higher quantities of the TthebZIP1 TF may be required after long metal-exposure periods. The highest *TthebZIP1* gene induction levels are detected in MTT5KD, GFPMTT5, and Cd-adap strains. These three strains have in common an increase in the *MTT1* gene expression levels as Cd-adap and MTT5KD strains have a higher number of copies for the *MTT1* gene or new *MTT1* paralogs comparing to the control strain (∼5× for Cd-adap and ∼3× for MTT5KD) (de Francisco et al., 2018). GFPMTT1 and GFPMTT5 strains have high *MTT1* expression levels under Cd^2+^ treatments (de Francisco et al., 2017). Therefore, a possible connection between *TthebZIP1* gene induction in response to Cd^2+^ or Pb^2+^ and *MTT1* gene expression levels, may be considered.

Further remarks on TthebZIP1 are as follows: (a)- the *TthebZIP1* gene is the first in the expression ranking in Cd-adap strain under the maximum cadmium tolerated concentration (MTC) (Supplementary Figure S2), and MTT1 is the *T. thermophila* MT with the highest binding preference to Cd^2+^ (Espart et al., 2015). (b)- In MTT5KD strain, with a considerably reduced *MTT5* gene copy number (de Francisco et al., 2018), *TthebZIP1* gene expression induction predominates in all metal treatments (at 1 and/or 24 h) (Supplementary Figure S2), and in this strain *MTT1* and *MTT5* genes are over-expressed (de Francisco et al., 2017). (c)- according to a microarray analysis (data from *Tetrahymena* Functional Genomic Database) (Xiong et al., 2013) this *TthebZIP1* gene is highly expressed during the first 2 h of *Tetrahymena* conjugation, and during the first 9 h of this sexual process all different MT genes are expressed. (d)- the *MTT1* gene promoter is the second *Tetrahymena* MT gene with the highest number of **TGA**(A/T/C)**TCA** motifs (AP-1 binding sites), to which the TthebZIP1 homodimers could be attached (de Francisco et al., 2016).

### TthebZIP2: A Transcription Factor Standing Up to Stressful Situations

In the majority of *T. thermophila* strains, *TthebZIP2* gene shows the third place in the basal expression raking, excepting for Cd-adap and MTT5KD strains in which this gene is in the first place, together with *TthebZIP4* (Supplementary Figure S2). Besides this, *TthebZIP2* gene is the one that achieves the highest induction levels, mainly after 24 h Cd^2+^ or Cu^2+^ exposures in Cu-adap and Cd-adap strains, respectively. Likewise, this gene (like *TthebZIP1*) is induced (fold-induction >2) in 20 different situations under metal stress and in different *T. thermophila* strains, about 7–13 times after 1 h or 24 h metal treatments (Supplementary Figure S2). The three metal adapted strains have the highest *TthebZIP2* gene expression induction values (Cd-adap > Pb-adap > Cu-adap) compared to the rest of the *TthebZIP* genes, after Cu^2+^ (in Cd-adap) or Cd^2+^ (in Cu- and Pb-adap strains) 24 h treatments (Figure 3 and Supplementary Table S7). Under these metal stress conditions, the MT genes *MTT2/4* (in Cd-adap) and *MTT5* (in Cu- and Pb-adap strains) are strongly over-expressed (de Francisco et al., 2017). In addition, the *TthebZIP2* gene is the only one induced in Pb-adap (Supplementary Figure S2), and in this strain is the *MTT5* gene the first one in the MT expression induction ranking, after any metal treatment (de Francisco et al., 2017).

The expression of *TthebZIP2* gene has also been detected during the conjugation process (*Tetrahymena* Functional Genomic Database) (Xiong et al., 2013), showing two maximum values; at 2–4 h (preceding to *MTT5, MTT1*, and *MTT3* gene expressions) and 10–12 h (preceding to the second maximum of *MTT5* gene expression) from the beginning of the conjugation.

*TthebZIP2* gene may intensely respond under stress situations when other bZIP TF isoforms are not able to respond so effectively. This gene seems to respond preferably after long metal exposure periods (24 h) and with some minor preference after short periods (1 h), and would also reinforce the cellular response in strains that are under higher stress conditions (metal-adapted, KO and/or KD strains). TthebZIP2 homodimers could also be indistinctly related to the induction of the five MT isoforms, activating one or another required MT gene in each stress situation.

### TthebZIP3 and TthebZIP4: Transcription Factors Related to *MTT5* Gene Expression

*TthebZIP3* gene shows the lowest basal expression levels comparing to the other *bZIP* genes in all analyzed strain (Supplementary Figure S2). However, the two KO strains suppressing the *MTT1* gene (MTT1KO and MTT1KO+MTT5KD) show the highest *TthebZIP3* basal expression levels; ∼6× and ∼7× higher than the SB1969 control strain, respectively. As previously suggested (de Francisco et al., 2017), *MTT5* gene may replace, in both strains, the loss of the completely eliminated *MTT1*, increasing its basal expression levels (∼8.5× and ∼59×, respectively), with regard to the control strain.

*TthebZIP3* shows similar induction patterns to *TthebZIP4* under metal-stress treatments (Figures 4, 5) and it is mainly expressed, but not exclusively, after Cd^2+^ (1 or 24 h) or Pb^2+^ (24 h) exposures (Supplementary Figure S2). This gene is also induced in Cd-adap (1 and 24 h Cu^2+^ treatment) and MTT5KD strain after Cu^2+^ (24 h), and it is the only expressed bZIP gene in MTT1KO+MTT5KD strain after Cu^2+^ (24 h) (Supplementary Figure S2). Regardless of the strain, the induction of the *TthebZIP3* gene appears 25 times in metal treated samples and so it is one of the bZIP genes that, together with *TthebZIP4*, is preferably induced under metal stress (Supplementary Figure S2). In general, it seems to have a more general response against metal stress conditions, being able to react after 1 and 24 h treatments.

During *T. thermophila* conjugation the *TthebZIP3* gene expression pattern overlaps or is close to those obtained from *MTT5* and *MTT3* genes, in addition, both genes (*TthebZIP3* and *MTT5*) have two maximum matching peaks during this sexual process.

*MTT5* gene is the mostly induced under Cd^2+^ or Pb^2+^ treatments in almost all analyzed *T. thermophila* strains (*MTT5* > *MTT1* > *MTT3* > *MTT2/4* consensus ranking), and under Cu^2+^ stress in some strains (Cu-adap, Pb-adap, and MTT5KD) (de Francisco et al., 2017). This essential MT gene has been considered as an “alarm” gene, that is over-expressed under metal stress (mainly Cd^2+^ and Pb^2+^) and promotes the expression of other MT genes (de Francisco et al., 2017). Likewise, a possible connection between *TthebZIP3* and *MTT5* expression may be considered, as both genes predominantly respond against different metals (mainly lead and cadmium).

*TthebZIP4* gene has the highest basal expression levels among the four *T. thermophila* bZIP genes independently of the analyzed strain (Supplementary Figure S2). It suggests the potential relevance of *TthebZIP4* gene as a gene expression regulator even under no stress conditions. This gene is induced in up to 23 different metal stress situations, being one of the most induced together with *TthebZIP3* (25 times). During *T. thermophila* conjugation *TthebZIP4* is also induced, and its maximum expression value is within the same conjugation temporal range (4–8 h) that the expression of *MTT5*, *MTT1*, *MTT3*, and *TthebZIP3* genes (as showed in *Tetrahymena* Functional Genomic Database) (Xiong et al., 2013).

*MTT5* gene has a promoter sequence duplicated, making it the MT gene with the largest number of AP-1 motifs (13 in total) (Diaz et al., 2007). This could involve a greater number of AP-1 TF molecules to increase their expression levels under stress conditions (Gutierrez et al., 2011). According to the scores obtained for all the possible *T. thermophila* AP-1 TF interactions, TthebZIP3 and TthebZIP4 are the most likely to form heterodimers. The formation of these heterodimers could be enhanced by the presence of the Cys residue present in each monomer (ThebZIP3 and TthebZIP4), which would form a thiolate bridge with the toxic metal (like Cd^2+^). Under no-stress conditions, the constitutive expression of *TthebZIP3* gene is the lowest while that of *TthebZIP4* is the highest in all *T. thermophila* analyzed strains (Supplementary Figure S2), and under these conditions the formation of TthebZIP4 homodimers could be favored. However, when TthebZIP3 TF is required (as when cells need to express the stress “alarm” *MTT5* gene), TthebZIP3/4 heterodimers may be formed after increasing the expression of the *TthebZIP3* gene (both genes are habitually induced under metal stress). Furthermore, against a stressful situation, the possibility of forming homo- or heterodimers increases cell response options as both conformations are normally related to the activation of different genes with putative different functions (de Francisco et al., 2018).

### A Global Outlook and a Model Integrating MT Gene Expression Regulation and the Four *T. thermophila* AP-1 Transcription Factors

AP-1 TFs are normally expressed under no-stress conditions but they are also induced against a large variety of stimulus that can cause cellular stress, such as: ultraviolet and ionizing radiations, oxidative stress, DNA damages, cytoskeleton reorganizations, etc. Even, in mammalian cells, these transcription factors are activated in response to neuronal depolarization events, the binding of antigens to B or T lymphocytes or cytokines detection, so these AP-1 TFs are key regulators in multiple physiological processes (de Francisco et al., 2018).

The MTCM1 motifs described in the promoter regions of the *T. thermophila* MT genes are putative AP-1 factor binding sites and their number of copies follows the ranking: *MTT5* (13) > >*MTT1* (6) > *MTT3* (2) = *MTT2/4* (2) (Diaz et al., 2007; de Francisco et al., 2016). Differences among the numbers of MTCM1 motifs are also related to MT gene expression levels against metal stress conditions and, presumably, to the number of AP-1 factors that can bind to their promoter regions to express these MT genes. Likewise, the ability of these AP-1 TFs to activate different genes may be related to post-translational modifications, the dimerization type or their association to different molecules acting as coactivators (Wisdom, 1999).

**FIGURE 7:**
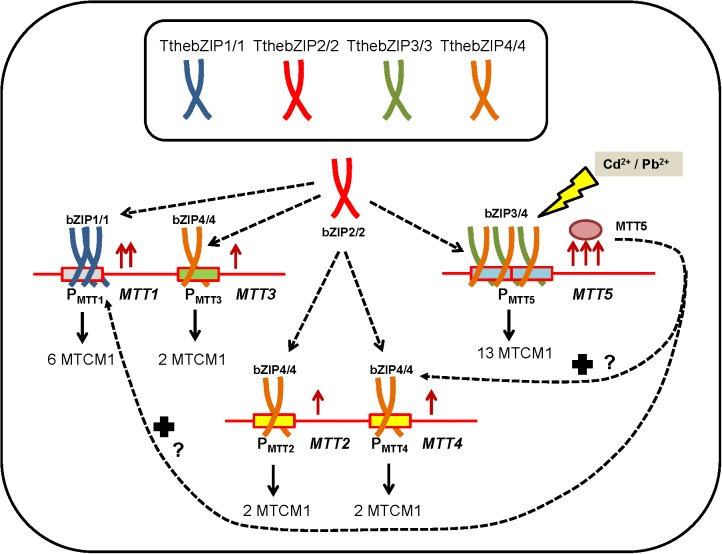
Hypothetical model representing the possible relationships among the different AP-1 TFs and MT genes of *T. thermophila* under metal-stress. P_MTT_, promoter region of each MT gene. MTCM1, metallothionein conserved motif 1. For further explanation see the text.

Results presented here provide some indirect evidences of the key role that the four *T. thermophila* AP-1 TFs may play in the MT gene expression regulation. The possible relationships among the different AP-1 TFs and MT genes of *T. thermophila* have been summarized in a hypothetical model (Figure 7).

The majority of these TthebZIP TFs seems to form homodimers (bZIP1/1, bZIP2/2, bZIP3/3, and bZIP4/4), while TthebZIP3 and TthebZIP4 may also form heterodimers (TthebZIP3/4) with the aim to promote *MTT5* gene expression (Figure 7). Moreover, considering the results, a possible preference of some TthebZIP TF to a specific MT promoter might exist, for instance; TthebZIP1 for *MTT1* promoter. Likewise, an unspecific TthebZIP TF with the ability to link to different MT gene promoters, such as TthebZIP2/2 or TthebZIP4/4 homodimers could also be considered (Figure 7). Finally, we have previously reported that high *MTT5* induction levels normally induce higher *MTT1* and *MTT2/4* expression levels (de Francisco et al., 2017), existing a connection among these three MT genes (*MTT5*, *MTT1*, and *MTT2/4*) and configuring a transcriptional regulatory network. Thus, MTT5 protein may interact, direct or indirectly, with TthebZIP gene promoters changing their expression levels and, therefore, having an additional function as a TthebZIP gene expression regulator (Figure 7). Of course, it is also possible that these TthebZIP TFs may also regulate the gene expression of other than the MT encoding genes.

From this study we can conclude the following main points:

(1) Four bZIP TFs have been described in all *Tetrahymena* analyzed species, showing a high conservation among them. Each AP-1 TF has its counterpart in the other three *Tetrahymena* species. This high evolutionary conservation may corroborate the functional relevance of these TFs in these microorganisms.
(2) Unusual high expression induction levels have been detected in some TthebZIP genes, and this induction seems to be associated to metallothionein gene expression during both metal stress and conjugation.
(3) TthebZIP1 homodimers might be related to *MTT1* gene expression regulation.

On the other hand, TthebZIP2 homodimers seem to be involved in a punctual reinforcement of MT genes expression under extreme metal stress conditions. TthebZIP3/4 heterodimers might be involved in the activation of the stress “alarm” *MTT5* gene. Which, in turn, increases the expression levels of *MTT1* and *MTT2/4* under metal stress. All these results are summarized in a hypothetical model of gene regulation involving *T. thermophila* MT genes, bZIP TFs and its connection to metal-stress cell response.

## Author’s Note

In memory of Silvia Atrian. Silvia Atrian passed away about a year ago. She was an excellent friend and Spanish scientific colleague. Her important research work on metallothioneins has represented a significant advance in this area. We dedicate this work to her memory.

## Author Contributions

PdF carried out all the qRT-PCR experiments, part of the *in silico* analysis of the bZIP TF sequences and drew up the first draft of the manuscript. FA carried out EMSA experiments and partial protein purification. AM-G assisted in statistical and *in silico* analysis. The project was conceived by JG who provided overall supervision of the study and the drafting of the manuscript. All authors read and approved the final manuscript.

## Conflict of Interest Statement

The authors declare that the research was conducted in the absence of any commercial or financial relationships that could be construed as a potential conflict of interest.

## Footnotes

1 http://pir.georgetown.edu/pirwww/search/comp_mw.shtml

2 http://2zip.molgen.mpg.de/

3 http://embnet.vital-it.ch/software/COILS_form.html

4 http://bioinf.cs.ucl.ac.uk/psipred/

5 http://compbio.cs.princeton.edu/bzip/

6 http://www.sbg.bio.ic.ac.uk/phyre2/html/page.cgi?id=index

7 http://pymol.org
