# Supplementary material for: AP-1 (bZIP) Transcription Factors as Potential Regulators of Metallothionein Gene Expression in Tetrahymena thermophila
Source: Front Genet. 2018 Oct 23;9:459. doi: 10.3389/fgene.2018.00459 (PMC6205968; doi:10.3389/fgene.2018.00459)
Supplement: Supplementary file 1 [file Table_1.DOCX]

**Table S1.** Primers used in this study

| **Primer** | **Sequence (5´- 3´)** | **Use (qRT-PCR)** |
| --- | --- | --- |
| TtACT1  TtACT2 | CTCTCTTTCTACCTTCCAAACT  AGGACCAGATTCATCATATTC | β-actin gene |
| TtAP1I-A  TtAP1I-B | AAGCTCTAAAGCTAGTGCAA  CATTTATGCTGATTCGATTT | *TthebZIP1* gene |
| TtAP1II-A  TtAP1II-B | TTGCATTTTGGTGATGTC  GCTCAAGAAAGCATTTACC | *TthebZIP2* gene |
| TtAP1III-A  TtAP1III-B | TGGAGCAACTGGAAGAAT  GAACGATTTATACATGTGCTTA | *TthebZIP3* gene |
| TtAP1IV-A  TtAP1IV-B | GAATAGCGGTAGTTAAG  GTTGAGTTATCTATCTGG | *TthebZIP4* gene |

**Table S2.** Quantitative RT-PCR standard-curve parameters

| **Strain** | **Gene** | **Slope** | **Efficiency (%)** | **Y-intercept** | **R^2(*)^** |
| --- | --- | --- | --- | --- | --- |
| **Control**  **SB1969** | *Β-actin* | -3.154 | 107.5 | 11.292 | 0.983 |
|  | *TthebZIP1* | -3.666 | 87.40 | 22.405 | 0.999 |
|  | *TthebZIP2* | -3.287 | 101.50 | 23.588 | 0.999 |
|  | *TthebZIP3* | -3.652 | 87.80 | 24.427 | 0.999 |
|  | *TthebZIP4* | -3.271 | 102.20 | 17.751 | 0.999 |
| **GFPMTT1** | *Β-actin* | -3.436 | 95.40 | 11.311 | 0.998 |
|  | *TthebZIP1* | -3.786 | 83.70 | 21.013 | 0.999 |
|  | *TthebZIP2* | -3.593 | 89.80 | 18.632 | 0.994 |
|  | *TthebZIP3* | -3.522 | 92.30 | 21.404 | 0.967 |
|  | *TthebZIP4* | -3.462 | 94.50 | 15.695 | 0.970 |
| **GFPMTT5** | *Β-actin* | -3.541 | 91.60 | 10.006 | 0.987 |
|  | *TthebZIP1* | -3.952 | 79.10 | 16.555 | 0.995 |
|  | *TthebZIP2* | -3.661 | 87.60 | 17.545 | 0.988 |
|  | *TthebZIP3* | -3.850 | 81.90 | 18.400 | 0.965 |
|  | *TthebZIP4* | -3.340 | 99.20 | 15.687 | 0.979 |
| **Cd-adap** | *Β-actin* | -3.393 | 97.10 | 15.985 | 0.999 |
|  | *TthebZIP1* | -3.543 | 91.50 | 19.192 | 0.997 |
|  | *TthebZIP2* | -3.535 | 91.80 | 23.039 | 0.996 |
|  | *TthebZIP3* | -3.607 | 89.00 | 26.952 | 0.988 |
|  | *TthebZIP4* | -3.499 | 93.10 | 15.266 | 0.979 |
| **Cu-adap** | *Β-actin* | -3.462 | 94.50 | 15.267 | 0.998 |
|  | *TthebZIP1* | -3.781 | 83.90 | 19.432 | 0.998 |
|  | *TthebZIP2* | -3.608 | 89.30 | 20.287 | 0.998 |
|  | *TthebZIP3* | -3.649 | 88.00 | 21.739 | 0.999 |
|  | *TthebZIP4* | -3.131 | 108.7 | 18.327 | 0.998 |
| **Pb-adap** | *Β-actin* | -3.401 | 96.80 | 13.248 | 0.999 |
|  | *TthebZIP1* | -3.514 | 92.60 | 24.737 | 0.999 |
|  | *TthebZIP2* | -3.260 | 102.6 | 22.087 | 0.991 |
|  | *TthebZIP3* | -3.015 | 115.0 | 26.134 | 0.958 |
|  | *TthebZIP4* | -3.131 | 108.7 | 18.327 | 0.998 |
| **MTT1KO** | *Β-actin* | -3.457 | 94.60 | 14.775 | 0.994 |
|  | *TthebZIP1* | -4.749 | 62.40 | 16.283 | 0.995 |
|  | *TthebZIP2* | -4.273 | 71.40 | 18.423 | 0.973 |
|  | *TthebZIP3* | -4.779 | 61.90 | 18.016 | 0.993 |
|  | *TthebZIP4* | -4.398 | 68.80 | 16.187 | 0.977 |
| **MTT5KD** | *Β-actin* | -3.592 | 89.90 | 10.963 | 0.987 |
|  | *TthebZIP1* | -3.361 | 98.40 | 18.833 | 0.991 |
|  | *TthebZIP2* | -4.709 | 63.10 | 18.143 | 0.997 |
|  | *TthebZIP3* | -4.469 | 67.00 | 19.290 | 0.983 |
|  | *TthebZIP4* | -3.904 | 80.40 | 20.681 | 0.959 |
| **MTT1KO + MTT5KD** | *Β-actin* | -3.280 | 102.8 | 11.972 | 0.996 |
|  | *TthebZIP1* | -4.099 | 75.40 | 18.532 | 0.999 |
|  | *TthebZIP2* | -3.400 | 96.80 | 20.739 | 0.971 |
|  | *TthebZIP3* | -3.575 | 90.00 | 21.954 | 0.942 |
|  | *TthebZIP4* | -3.584 | 91.10 | 18.633 | 0.954 |

(*): correlation coefficient. Efficiency (E) is calculated from the slope value of the standard curve: E = 10^(-1/slope)-1^

**Table S3.** Oligonucleotides used in EMSA experiments and partial protein purification binding to MTCM1 motifs

| **Oligonucleotide** | **Sequence (5´- 3´)** |
| --- | --- |
| MTCMFw | Dig-AATTTATG**TGATTCA**TGAGTCTAGC |
| MTCMRev | Dig-GCTAGACTCA**TGAATCA**CATAAATT |
| MTCMAB2 | AATTTATG**TGATTCA**TGAGTCTAGCAATTTATG**TGATTCA**TGAGTCTAGC TTAAATAC**ACTAAGT**ACTCAGATCGTTAAATAC**ACTAAGT**ACTCAGATCG |

Dig: digoxigenin labelled 5´end. In bold type TGANTCA motifs are indicated.

**Table S4.** Protein-DNA binding reactions to determine optimal conditions for EMSA experiments

| **Reaction component** | **Reactions (volume in μl)**  **1 2 3 4 5 6** |
| --- | --- |
| Nuclear extract (3-75 μg/μl)  Binding buffer  Poly(dI-dC) (1μg/μl)  MgCl_2_ (25 mM)  Dig-DNA probe (50 fmoles/μl)  H_2_O | - 2 4 6 8 12  12 10 8 6 4 2  1 1 1 1 1 1  2 2 2 2 2 2  1 1 1 1 1 1  4 4 4 4 4 2 |

**Table S5.** Access numbers defined in GenBank (NCBI), *Tetrahymena, Oxytricha* or *Stentor* Genome Databases for all bZIP transcription factors used in this study

| **Specie** | **Name of the transcription factor** | **Access number** |
| --- | --- | --- |
| ***T. thermophila*** | TthebZIP1 | TTHERM_00442410 |
|  | TthebZIP2 | TTHERM_00245720 |
|  | TthebZIP3 | TTHERM_00442930 |
|  | TthebZIP4 | TTHERM_00349090 |
| ***T. borealis*** | TborbZIP1 | EI9_12572.2 |
|  | TborbZIP2 | EI9_09817.1 |
|  | TborbZIP3 | EI9_00516.1 |
|  | TborbZIP4 | EI9_12630.1 |
| ***T. malaccensis*** | TmalabZIP1 | EIA_12351.2 |
|  | TmalabZIP2 | EIA_03662.2 |
|  | TmalabZIP3 | EIA_12297.2 |
|  | TmalabZIP4 | EIA_09172.2 |
| ***T. elliotti*** | TellibZIP1 | EI7_15490.3 |
|  | TellibZIP2 | EI7_01112.3 |
|  | TellibZIP3 | EI7_15548 |
|  | TellibZIP4 | EI7_14928.3 |
| ***Oxytricha sp.*** | OxybZIP | Contig10614.0.g91 |
| ***Stentor coeruleus*** | ScoebZIP1 | SteCoe­­_2470 |
|  | ScoebZIP2 | SteCoe_2759 |
|  | ScoebZIP3 | SteCoe_9041 |
|  | ScoebZIP4 | SteCoe_16647 |
|  | ScoebZIP5 | SteCoe_26280 |
|  | ScoebZIP6 | SteCoe_29990 |
|  | ScoebZIP7 | SteCoe_38490 |
| ***Homo sapiens*** | FOS | CAG47063 |
| ***Caenorhabditis elegans*** | FOS-1 | NP_001033481 |
| ***Mus musculus*** | JUN-B | NP_032442 |
| ***Rattus norvegicus*** | ATF3 | NP_037044 |

**Table S6.** Scores defining the most probable interactions between different *Tetrahymena* AP-1 monomers

| ***T. thermophila*** | | | | |
| --- | --- | --- | --- | --- |
| **Interaction** | **TthebZIP1** | **TthebZIP2** | **TthebZIP3** | **TthebZIP4** |
| **TthebZIP1** | **43.89** |  |  |  |
| **TthebZIP2** | -15.82 | **32.64** |  |  |
| **TthebZIP3** | 10.38 | -7.16 | **29.32** |  |
| **TthebZIP4** | 8.63 | 0.25 | **23.14** | **20.33** |
| ***T. borealis*** | | | | |
| **Interaction** | **TborbZIP1** | **TborbZIP2** | **TborbZIP3** | **TborbZIP4** |
| **TborbZIP1** | **47.90** |  |  |  |
| **TborbZIP2** | 9.93 | **23.57** |  |  |
| **TborbZIP3** | -13.96 | 8.01 | **23.72** |  |
| **TborbZIP4** | 11.67 | **23.20** | -4.52 | **29.32** |
| ***T. elliotti*** | | | | |
| **Interaction** | **TellibZIP1** | **TellibZIP2** | **TellibZIP3** | **TellibZIP4** |
| **TellibZIP1** | **43.89** |  |  |  |
| **TellibZIP2** | -16.82 | **32.64** |  |  |
| **TellibZIP3** | -1.56 | -7.16 | **29.32** |  |
| **TellibZIP4** | 8.63 | 0.25 | **23.14** | **20.33** |
| ***T. malaccensis*** | | | | |
| **Interaction** | **TmalabZIP1** | **TmalabZIP2** | **TmalabZIP3** | **TmalabZIP4** |
| **TmalabZIP1** | **43.89** |  |  |  |
| **TmalabZIP2** | -2.97 | **46.99** |  |  |
| **TmalabZIP3** | 10.32 | -2.35 | **29.32** |  |
| **TmalabZIP4** | 8.63 | -3.30 | **23.14** | **20.33** |

Scores for the formation of identical (homodimers) or different monomers (heterodimers). Higher scores indicate to most probable interactions (marked in red). Negative values are considered as no optimal interactions.

**Table S7.** Relative gene expression induction values obtained by qRT-PCR in the four *T. thermophila* AP-1genes

| ***TthebZIP1* gene** | | | | | | | | | |
| --- | --- | --- | --- | --- | --- | --- | --- | --- | --- |
| **Strain** | **Treatment** | | **Cd 1h** | **Cd 24h** | **Cu 1h** | **Cu 24h** | **Pb 1h** | **Pb 24h** | **MTC** |
| **Control SB1969** | | | **2.06 ± 0.59** | **2.84 ± 0.70** | **2 .00 ± 0.36** | 1.58 ± 0.31 | 1.19 ± 0.20 | **5.7 ± 0.39** | - |
| **Cd-adap** | | | 1.52 ± 0.62 | **4.92 ± 0.28** | **2.68 ± 0.14** | **2.21 ± 0.29** | 0.07 ± 0.02 | 0.60 ± 0.05 | **5.48 ± 0.48** |
| **Cu-adap** | | | **2.68 ± 0.62** | **2.90 ± 0.68** | 0.80 ± 0.16 | 0.44 ± 0.10 | 0.01 ± 0.004 | 0.15 ± 0.04 | 0.46 ± 0.07 |
| **Pb-adap** | | | 0.38 ± 0.03 | 0.46 ± 0.085 | 0.38 ± 0.02 | 1.004 ± 0.15 | 0.10 ± 0.01 | 0.04 ± 0.002 | 0.26 ± 0.09 |
| **GFPMTT5** | | | **2.70 ± 0.32** | **7.44 ± 1.37** | 1.14 ± 0.085 | **2.71 ± 0.42** | 0.15 ± 0.004 | **2.32 ± 0.32** | - |
| **GFPMTT1** | | | 1.16 ± 0.25 | 1.37 ± 0.27 | 0.67 ± 0.10 | 0.59 ± 0.15 | 0.01 ± 0.002 | 0.30 ± 0.06 | - |
| **MTT1KO** | | | **3.18 ± 0.08** | 1.63 ± 0.13 | 1.69 ± 0.25 | **3.03 ± 0.22** | 1.99 ± 0.27 | 1.46 ± 0.45 | - |
| **MTT5KD** | | | **15.18 ± 2.98** | **17.97 ± 5.50** | 1.09 ± 0.11 | **4.47 ± 0.89** | 1.11 ± 0.03 | **9.37 ± 1.49** | - |
| **MTT1KO+MTT5KD** | | | 1.06 ± 0.09 | 1.90 ± 0.12 | 0.91 ± 0.06 | 1.15 ± 0.14 | 1.11 ± 0.18 | **2.26 ± 0.24** | - |
| ***TthebZIP2* gene** | | | | | | | | | |
| **Strain** | **Treatment** | | **Cd 1h** | **Cd 24h** | **Cu 1h** | **Cu 24h** | **Pb 1h** | **Pb 24h** | **MTC** |
| **Control SB1969** | | | 1.04 ± 0.33 | **2.19 ± 0.40** | 1.20 ± 0.24 | 0.99 ± 0.13 | 0.96 ± 0.17 | **5.37 ± 0.45** | - |
| **Cd-adap** | | | 1.01 ± 0.39 | **2.45 ± 0.13** | **2.30 ± 0.05** | **63.43 ± 7.36** | 0.81 ± 0.3 | **2.05 ± 0.16** | 1.12 ± 0.13 |
| **Cu-adap** | | | 1.77 ± 0.42 | **16.70 ± 4.01** | 0.85 ± 0.13 | 0.28 ± 0.05 | 0.11 ± 0.03 | 0.29 ± 0.04 | 0.35 ± 0.06 |
| **Pb-adap** | | | 1.78 ± 0.10 | **35.03 ± 5.96** | **2.75 ± 0.26** | 0.27 ± 0.04 | **3.53 ± 0.21** | 0.91 ± 0.03 | 0.90 ± 0.34 |
| **GFPMTT5** | | | **2.91 ± 0.61** | **8.25 ± 1.79** | 1.91 ± 0.26 | 0.89 ± 0.18 | 0.79 ± 0.13 | 1.87 ± 0.44 | - |
| **GFPMTT1** | | | 1.54 ± 0.36 | **15.01 ± 2.48** | **3.77 ± 0.32** | 0.78 ± 0.24 | 0.15 ± 0.007 | 1.42 ± 0.31 | - |
| **MTT1KO** | | | **2.81 ± 0.31** | **2.95 ± 0.07** | 1.29 ± 0.21 | **3.69 ± 0.27** | **3.12 ± 0.40** | 1.30 ± 0.40 | - |
| **MTT5KD** | | | 0.84 ± 0.15 | 1.02 ± 0.30 | 0.61 ± 0.06 | 1.06 ± 0.24 | 0.65 ± 0.001 | 0.84 ± 0.14 | - |
| **MTT1KO+MTT5KD** | | | 1.06 ± 0.08 | **8.06 ± 0.41** | 1.02 ± 0.07 | 0.96 ± 0.12 | 1.05 ± 0.06 | **2.18 ± 0.19** | - |
| ***TthebZIP3* gene** | | | | | | | | | |
| **Strain** | | **Treatment** | **Cd 1h** | **Cd 24h** | **Cu 1h** | **Cu 24h** | **Pb 1h** | **Pb 24h** | **MTC** |
| **Control SB1969** | | | **2.10 ± 0.70** | **2.68 ± 0.49** | 1.44 ± 0.33 | 1.44 ± 0.35 | 1.17 ± 0.40 | **8.25 ± 0.64** | - |
| **Cd-adap** | | | 1.55 ± 0.46 | **9.29 ± 0.61** | **4.97 ± 1.78** | **2.23 ± 0.51** | 0.27 ± 0.08 | **2.08 ± 0.30** | **4.55 ± 1.28** |
| **Cu-adap** | | | **3.61 ± 0.87** | **7.21 ± 1.82** | 1.05 ± 0.14 | 0.84 ± 0.15 | 0.08 ± 0.02 | 0.35 ± 0.04 | 0.62 ± 0.09 |
| **Pb-adap** | | | 0.44 ± 0.05 | 1.30 ± 0.36 | 0.49 ± 0.03 | 0.68 ± 0.17 | 0.25 ± 0.01 | 0.12 ± 0.05 | 0.38 ± 0.14 |
| **GFPMTT5** | | | **5.99 ± 1.06** | **6.32 ± 0.84** | 1.66 ± 0.36 | **2.20 ± 0.48** | 0.34 ± 0.02 | **2.21 ± 0.45** | - |
| **GFPMTT1** | | | **2.46 ± 0.59** | 1.42 ± 0.34 | 0.20 ± 0.03 | 0.62 ± 0.13 | 0.07 ± 0.02 | 0.43 ± 0.08 | - |
| **MTT1KO** | | | **2.40 ± 0.06** | **2.69 ± 0.06** | 1.23 ± 0.20 | 2.60 ± 0.18 | **2.10 ± 0.27** | 1.76 ± 0.54 | - |
| **MTT5KD** | | | **5.56 ± 0.97** | **6.33 ± 1.76** | 1.03 ± 0.09 | **3.33 ± 0.66** | 0.88 ± 0.09 | **3.45 ± 0.55** | - |
| **MTT1KO+MTT5KD** | | | 1.10 ± 0.07 | **7.10 ± 0.35** | 0.63 ± 0.03 | **2.27 ± 0.29** | 0.95 ± 0.02 | **3.40 ± 0.30** | - |
| ***TthebZIP4* gene** | | | | | | | | | |
| **Strain** | | **Treatment** | **Cd 1h** | **Cd 24h** | **Cu 1h** | **Cu 24h** | **Pb 1h** | **Pb 24h** | **MTC** |
| **Control SB1969** | | | **2.15 ± 0.96** | **2.39 ± 0.36** | **2.2 ± 0.12** | **4.55 ± 0.18** | 1.11 ± 0.23 | 1.34 ± 0.37 | - |
| **Cd-adap** | | | 1.86 ± 0.49 | **8.54 ± 0.38** | **8.53 ± 0.22** | **5.37 ± 0.33** | 0.17 ± 0.04 | **2.38 ± 0.18** | **3.13 ± 0.20** |
| **Cu-adap** | | | **2.84 ± 0.64** | **5.14 ± 1.26** | **2.12 ± 0.19** | 0.96 ± 0.18 | 0.01 ± 0.003 | 0.20 ± 0.016 | 0.54 ± 0.08 |
| **Pb-adap** | | | 0.68 ± 0.05 | 1.03 ± 0.09 | 0.49 ± 0.03 | 0.32 ± 0.16 | 0.10 ± 0.005 | 0.12 ± 0.006 | 0.45 ± 0.17 |
| **GFPMTT5** | | | 1.47 ± 0.16 | **2.31 ± 1.09** | **4.22 ± 1.60** | 0.38 ± 0.13 | 0.25 ± 0.01 | 1.20 ± 0.17 | - |
| **GFPMTT1** | | | 0.75 ± 0.19 | 0.77 ± 0.11 | 0.69 ± 0.22 | 0.70 ± 0.14 | 0.05 ± 0.002 | 0.36 ± 0.04 | - |
| **MTT1KO** | | | **2.45 ± 0.34** | **2.85 ± 0.09** | 1.68 ± 0.25 | **2.68 ± 0.20** | **2.05 ± 0.30** | 1.43 ± 0.45 | - |
| **MTT5KD** | | | **5.67 ± 1.002** | **4.92 ± 1.37** | 1.11 ± 0.11 | **3.28 ± 0.67** | 1.43 ± 0.03 | **3.01 ± 0.48** | - |
| **MTT1KO+MTT5KD** | | | 0.95 ± 0.07 | **3.50 ± 0.12** | 0.58 ± 0.03 | 1.25 ± 0.28 | 0.63 ± 0.07 | 1.30 ± 0.23 | - |

(-): Data not obtained. MTC: maximum tolerated concentration in metal-adapted strains. The average values ± standard deviations from two or three independent experiments are shown. Normalization of the gene expression was carried out using the β-actin as an endogenous control gene. Induction values higher than the minimum considered induction value (≥ 2) are in red.

**Table S8.** C_t_ values obtained for each *T. thermophila* AP-1 gene under control conditions (no metal treatment)

| **Gene** | **Strain** | **Control** | **Cd-adap** | **Cu-adap** | **Pb-adap** |  |
| --- | --- | --- | --- | --- | --- | --- |
| ***ACTIN*** | | 14.90 | 15.80 | 15.20 | 15.20 |  |
| ***TthebZIP1*** | | 23.67 | 23.78 | 22.33 | 22.07 |  |
| ***TthebZIP2*** | | 23.51 | 23.19 | 23.34 | 23.54 |  |
| ***TthebZIP3*** | | 25.86 | 27.23 | 24.98 | 25.06 |  |
| ***TthebZIP4*** | | 21.10 | 23.30 | 21.80 | 20.50 |  |
| **Gene** | **Strain** | **GFPMTT1** | **GFPMTT5** | **MTT1KO** | **MTT5KD** | **MTT1KO+MTT5KD** |
| ***ACTIN*** | | 14.80 | 15.10 | 13.00 | 15.00 | 14.10 |
| ***TthebZIP1*** | | 20.98* | 23.79 | 22.40 | 25.80 | 21.70 |
| ***TthebZIP2*** | | 22.66 | 24.42 | 22.70 | 23.4 | 22.80 |
| ***TthebZIP3*** | | 24.25 | 26.25 | 23.30* | 26.60 | 23.00* |
| ***TthebZIP4*** | | 19.80 | 21.25 | 20.70 | 23.60 | 20.70 |

*Actin* gene was used as an endogenous control in qRT-PCR assays. (*) C_t_ values considerably lower (with a difference of at least 2 cycles) than those obtained in the SB1969 control strain, so showing higher basal expression levels for a particular gene in a specific strain.


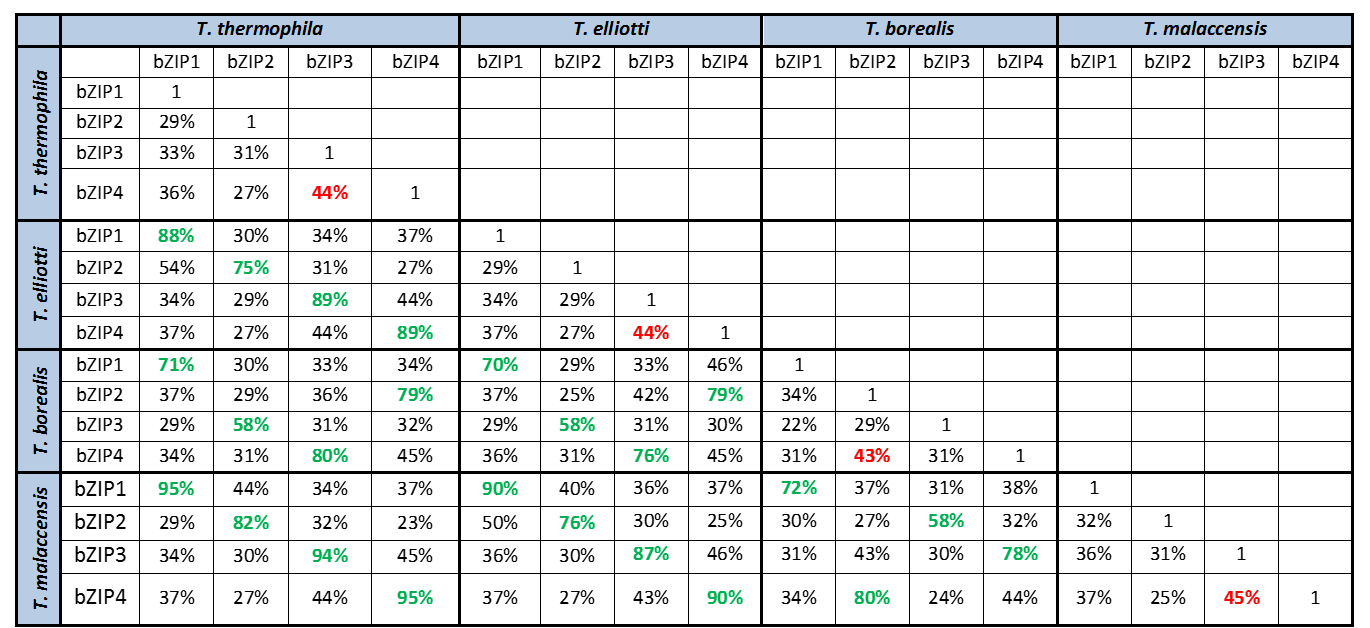


**Table S9.** Identity matrix of all bZIP amino acid sequences from the four *Tetrahymena* species

The highest identity percentages among the four bZIP transcription factor in each species are marked in red. The highest identity percentages for each bZIP transcription factor between two different species are marked in green.
