# Supplementary material for: AP-1 (bZIP) Transcription Factors as Potential Regulators of Metallothionein Gene Expression in Tetrahymena thermophila
Source: Front Genet. 2018 Oct 23;9:459. doi: 10.3389/fgene.2018.00459 (PMC6205968; doi:10.3389/fgene.2018.00459)
Supplement: Supplementary file 2 [file Table_2.DOCX]

**Supplementary Material Figure-captions**

**Figure S1**. Putative coiled-coil motifs defined by COILS program for all the *Tetrahymena* bZIP TFs. Each graph shows amino acid regions able to interact with other bZIP protein (monomer) to form coiled-coil structures and their probability of being dimerization regions. Red arrows mark regions with a higher probability for dimerizing and forming coiled-coil structures. Tthe: T. thermophila; Tbor: T. borealis; Tmala: T. malaccensis; Telli: T. elliotti.

**Figure S2**. Rankings of basal expression levels (no metal exposure) and induction values (Cd2+, Cu2+ or Pb2+ treatments) obtained from the four AP-1 genes for each *T. thermophila* strain analyzed. Only induction values > 2 are considered.

**Figure S3**. EMSA assays. (**A**): Chemilumigram obtained after incubation with Dig-labelled MTCM1 oligonucleotide under increasing concentrations of *T. thermophila* macronuclear protein (Mac prot) extracts (isolated from 0.9 μM Cd2+ treated cultures during 2h) and different reaction conditions (lanes 2-7). Poly(dI-dC) oligonucleotide acted as an unspecific competitor. DNA size standard (MVI digoxigenin-labelled, Roche) (lane C). (**B**): Specific-competition assays. Non-Dig-labelled MTCM1 (specific competitor at 50x, 100x or 200x concentration) (lanes 5-7). Macronuclear protein extracts from Cd-treated cultures during 30 min. (lanes 1 and 2), or 2h (lanes 3-7). *Escherichia coli* protein extract (lane N). Control (without Mac prot) (lanes 0).

**Figure S4**. Southwestern blotting chemilumigram. Macronuclear extracts (100 μg) from T. thermophila 1.5 μM Cd-treated cultures during 2h (lanes 1 and 2). Red arrows indicate positive bands. Protein size standard (Broad Range, Bio-Rad) (lane M). Negative control (100 μg BSA protein) (lane C).
