# Supplementary figures and images for: AP-1 (bZIP) Transcription Factors as Potential Regulators of Metallothionein Gene Expression in Tetrahymena thermophila
Source: Front Genet. 2018 Oct 23;9:459. doi: 10.3389/fgene.2018.00459 (PMC6205968; doi:10.3389/fgene.2018.00459)

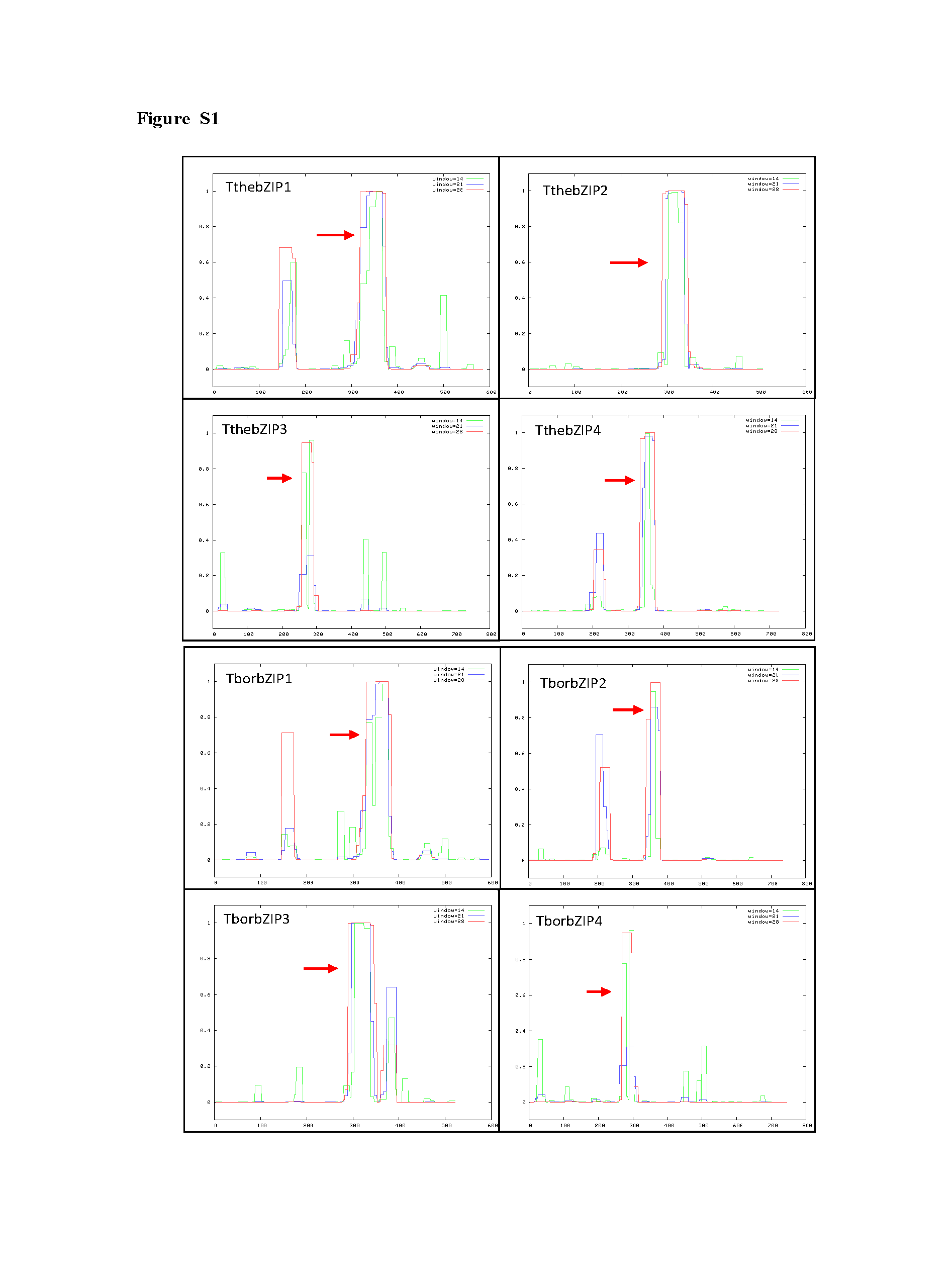

Supplement: Supplementary file 3 [file Image_1.TIFF]

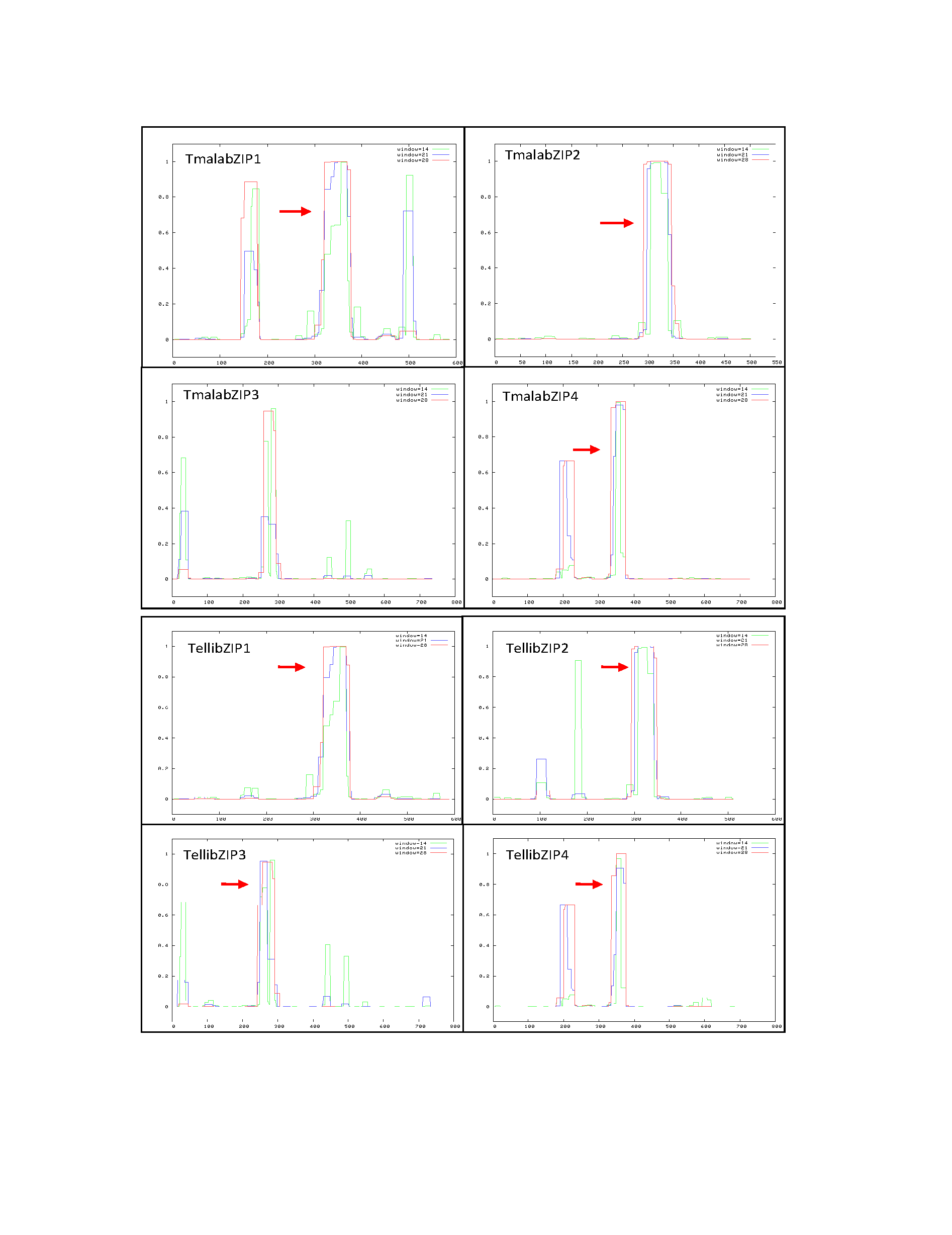

Supplement: Supplementary file 4 [file Image_2.TIFF]

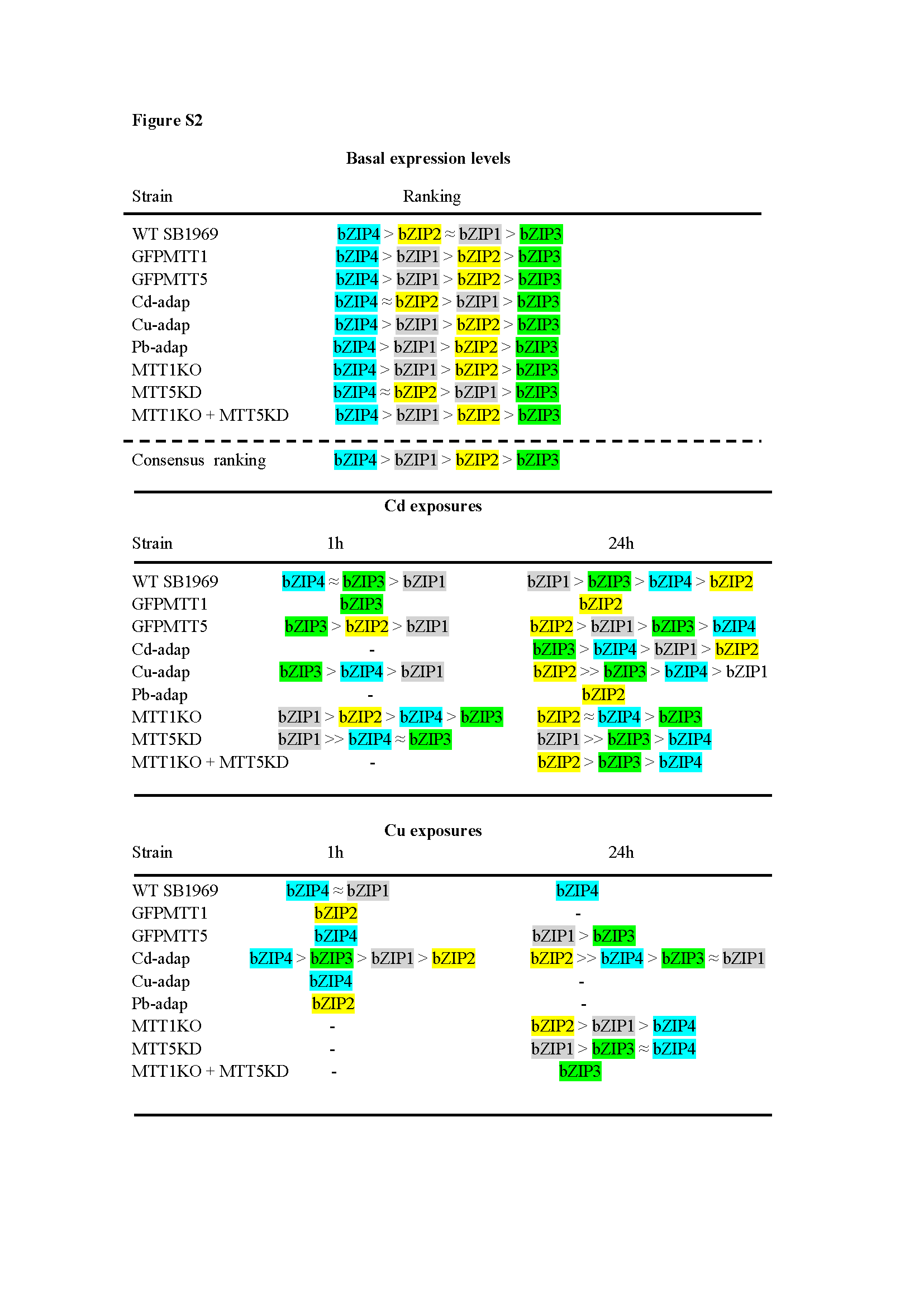

Supplement: Supplementary file 5 [file Image_3.TIFF]

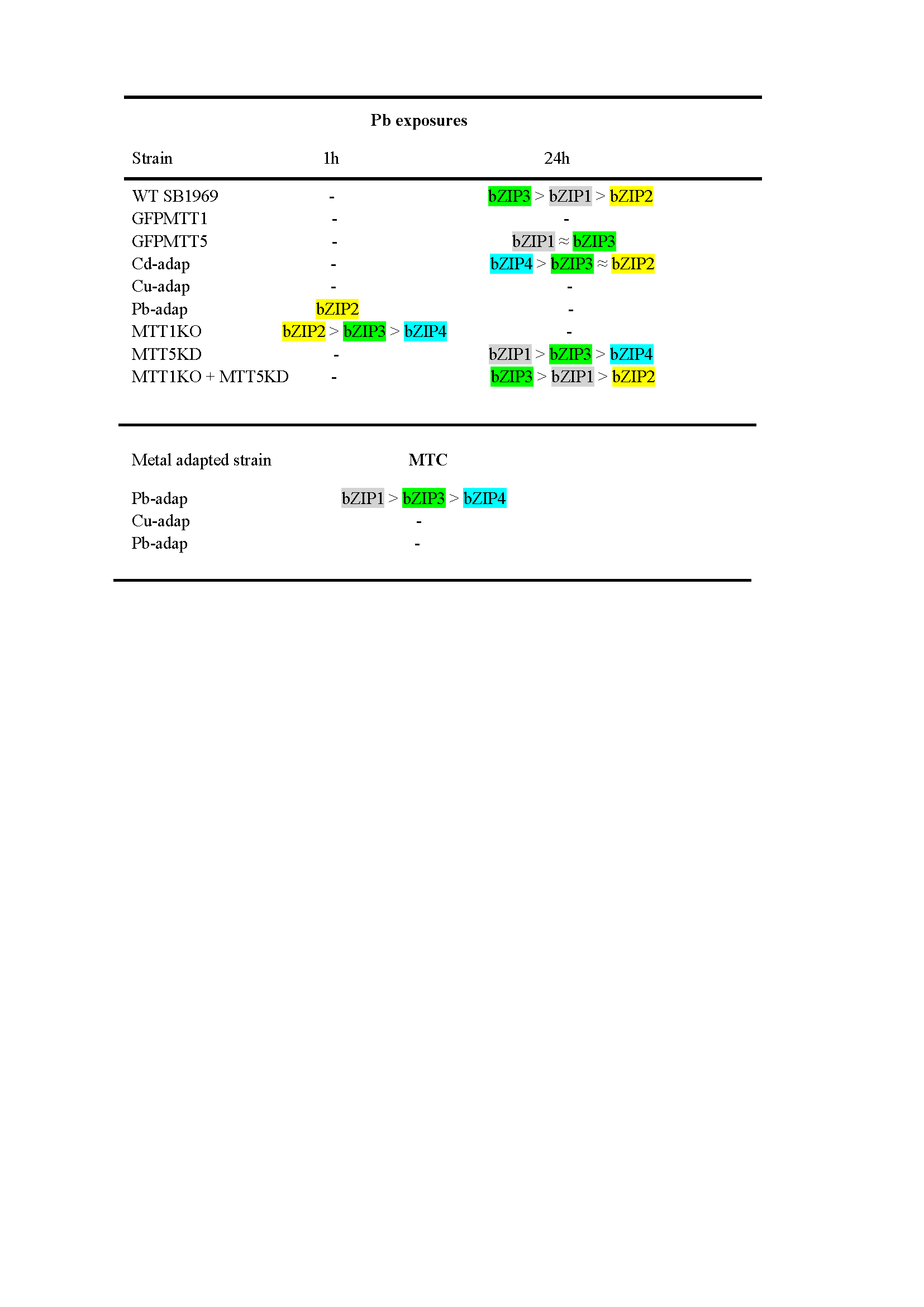

Supplement: Supplementary file 6 [file Image_4.TIFF]

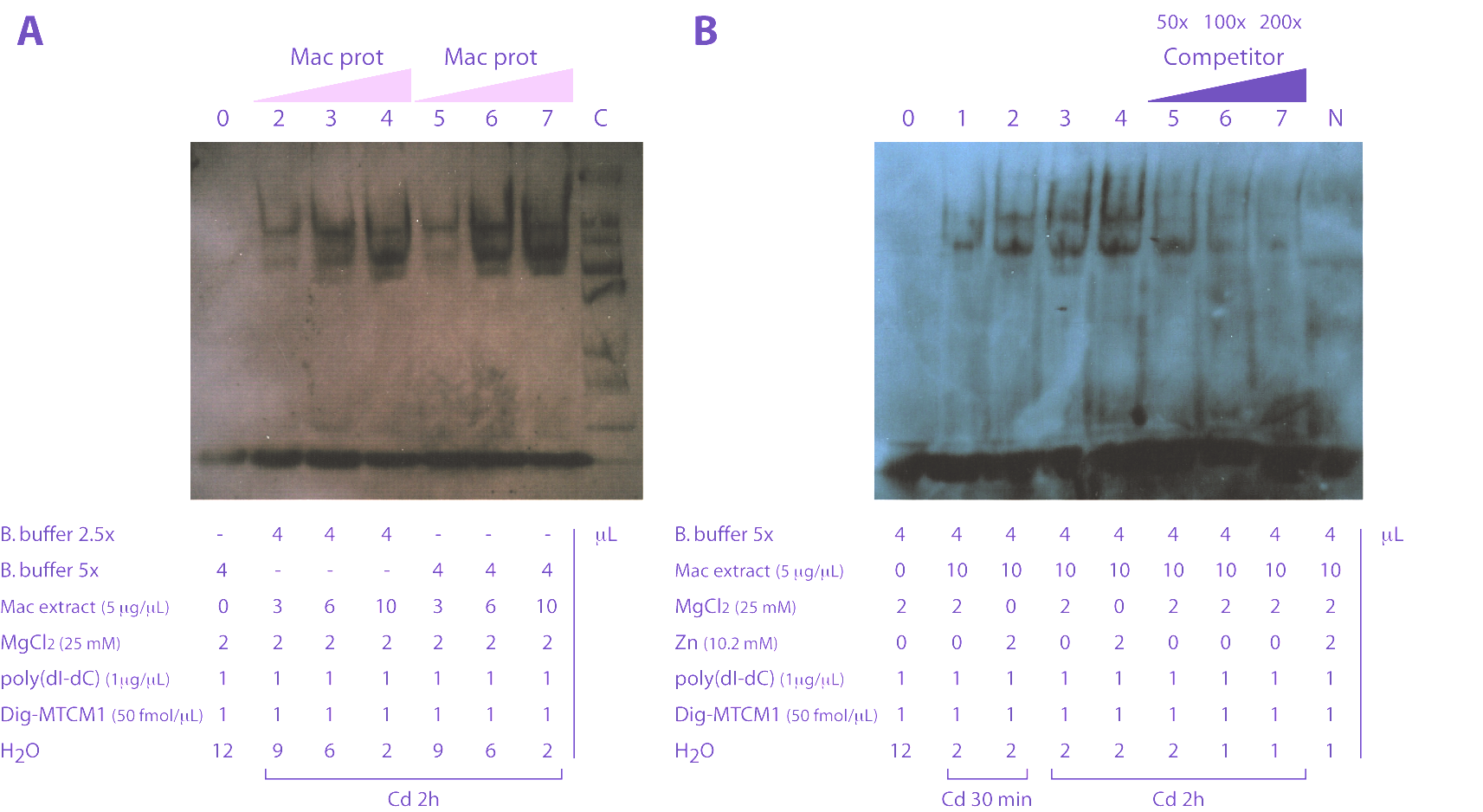

Supplement: Supplementary file 7 [file Image_5.TIF]

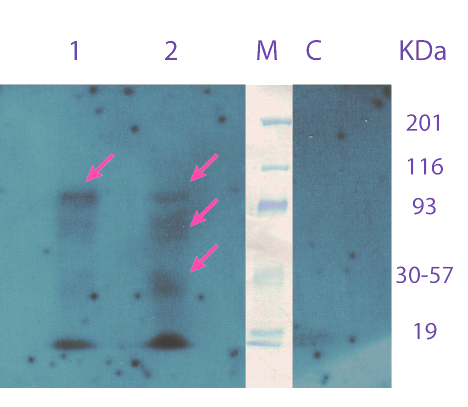

Supplement: Supplementary file 8 [file Image_6.TIF]
